# Networks of myelin covariance

**DOI:** 10.1002/hbm.23929

**Published:** 2017-12-21

**Authors:** Lester Melie‐Garcia, David Slater, Anne Ruef, Gretel Sanabria‐Diaz, Martin Preisig, Ferath Kherif, Bogdan Draganski, Antoine Lutti

**Affiliations:** ^1^ LREN, Department of Clinical Neurosciences Lausanne University Hospital (CHUV) Switzerland; ^2^ Department of Psychiatry Lausanne University Hospital (CHUV) Switzerland; ^3^ Max‐Planck‐Institute for Human Cognitive and Brain Sciences Leipzig Germany

**Keywords:** aging, brain connectivity, graph theory, magnetization transfer, myelin, myelination, precuneus, quantitative MRI, structural network

## Abstract

Networks of anatomical covariance have been widely used to study connectivity patterns in both normal and pathological brains based on the concurrent changes of morphometric measures (i.e., cortical thickness) between brain structures across subjects (Evans, 2013). However, the existence of networks of microstructural changes within brain tissue has been largely unexplored so far. In this article, we studied in vivo the concurrent myelination processes among brain anatomical structures that gathered together emerge to form nonrandom networks. We name these “networks of myelin covariance” (*Myelin‐Nets*). The *Myelin‐Nets* were built from quantitative Magnetization Transfer data—an in‐vivo magnetic resonance imaging (MRI) marker of myelin content. The synchronicity of the variations in myelin content between anatomical regions was measured by computing the Pearson's correlation coefficient. We were especially interested in elucidating the effect of age on the topological organization of the *Myelin‐Nets*. We therefore selected two age groups: Young‐Age (20–31 years old) and Old‐Age (60–71 years old) and a pool of participants from 48 to 87 years old for a *Myelin‐Nets* aging trajectory study. We found that the topological organization of the *Myelin‐Nets* is strongly shaped by aging processes. The global myelin correlation strength, between homologous regions and locally in different brain lobes, showed a significant dependence on age. Interestingly, we also showed that the aging process modulates the resilience of the *Myelin‐Nets* to damage of principal network structures. In summary, this work sheds light on the organizational principles driving myelination and myelin degeneration in brain gray matter and how such patterns are modulated by aging.

## 1 INTRODUCTION

Brain myelination is the process that takes place when myelin, which is made up of fatty lipids (principally cholesterol and proteins), accumulates around nervous fibers. Despite myelin density being sparser in gray matter compared to white matter, this tissue component plays an important role in insulating local axons that connects neighboring and distant areas. It introduces a boost in axonal conduction velocities making it possible for brain areas to interchange information in optimal synchrony. It has been proved that the impaired cognitive ability, disorganized thinking, mood disorders or hallucinations associated with psychiatric illness (i.e., Schizophrenia) might result from slowed or desynchronized impulse conduction between cortical regions due to deficiencies of myelin wrapping the axons (Fields, 2008; Gootjes et al., 2004; Kujala, Portin, & Ruutiainen, 1997). Experimental evidence shows that tissue myelination may be tuned by experience to satisfy requirements of synchronicity in neural circuits and achieve optimal mental performance and learning (Fields, 2008). Myelination is also the subject of profound changes across the lifespan with gray matter myelination following inverted‐U shaped trajectories with age (Grydeland, Walhovd, Tamnes, Westlye, & Fjell, 2013) whose characteristics exhibit specific anatomical distribution patterns (Dean et al., 2016). So far, however, the important question of the interactions between local cortical myelination changes remains largely unexplored. Do myelin changes occur independently across brain regions? Is myelination in one region related to or modulated by variations in other regions? Are synchronized myelination changes across the cortical mantle topologically organized? Could common underlying factors (e.g., genetics) be driving these concurrent myelin fluctuations? Our paper is precisely motivated by such questions.

The analysis of the covariance between regional estimates of morphometric features derived from magnetic resonance images (MRI) is well established (Andrews, Halpern, & Purves, 1997; Mechelli, Friston, Frackowiak, & Price, 2005). Recent studies have investigated in detail the topological organization of the brain (He, Chen, & Evans, 2007; Sanabria‐Diaz et al., 2010) to provide evidence for a network of anatomical covariance in the healthy and diseased brain (Alexander‐Bloch, Giedd, & Bullmore, 2013; Evans, 2013). A number of studies have shown strong age‐related effects on the number and strength of the interhemispheric correlations in cortical thickness and three key network topological properties: characteristic path length, clustering index, and local efficiency (Chen, He, Rosa‐Neto, Gong, & Evans, 2011; Wu et al., 2012; Yang, Tsai, Liu, Huang, & Lin, 2016; Zhu et al., 2012).

Morphological covariance studies are based on macroscopic features of the brain and do not provide an insight into the concurrent changes taking place at the microscopic scale within brain tissue. The latter changes are of primary interest in neuroscience but are largely intractable from the most widely used types of anatomical MRI scans (e.g., T1‐weighted, T2‐weighted). A recent study has shown that microscopic changes within brain tissue could lead to the spurious detection of apparent morphological change (Lorio et al., 2016). New methods have emerged based on the ratio of T1‐weighted and T2‐weighted images (Glasser and Van Essen, 2011) that provide an insight into the biological processes underlying brain tissue changes. However multiple microscopic properties of the brain may impact ratio estimates, complicating the interpretability of observed findings. Quantitative MRI (qMRI) addresses the limitations of standard MRI anatomical data by providing quantitative estimates of the MRI parameters that drive signal intensities in an MRI image. The correlation between these estimates and microscopic features of brain tissue has motivated their use as in vivo markers of microstructure (Lutti, Dick, Sereno, & Weiskopf, 2014). qMRI data is corrected for the sources of artifact that affect standard anatomical MRI images, leading to enhanced reproducibility and sensitivity to physiological brain changes (Weiskopf et al., 2013). Because myelin and iron concentrations are the main contributors to qMRI estimates (Fukunaga et al., 2010; Schmierer et al., 2007; Stüber et al., 2014), the qMRI changes reported in neuroscience applications have mainly been attributed to iron deposition and demyelination processes, in line with histological findings (Callaghan et al., 2014; Draganski et al., 2011). qMRI also allows the in vivo delineation of the heavily myelinated boundaries of visual (Sereno, Lutti, Weiskopf, & Dick, 2013) and primary auditory (Dick et al., 2012) areas.

This study is based on the MRI parameter, Magnetization Transfer (MT) (Helms, Dathe, Kallenberg, & Dechent, 2008b), which exhibits a high level of specificity toward tissue myelination (Callaghan et al., 2014; Helms, Draganski, Frackowiak, Ashburner, & Weiskopf, 2009; Lorio et al., 2014; Lorio et al., 2016). The MT mechanism is based on the exchange of magnetization between free water and protons bound to macromolecules. MT values are mainly driven by the local macromolecule density and the amount of water in close proximity with these macromolecules. Amongst the macromolecules involved, myelin cholesterol has been suggested as a major contributor to magnetization transfer (Koenig, Brown, Spiller, & Lundbom, 1990; Koenig, 1991).

Recently, using MT maps, Hunt et al. (2016) evidenced the presence of myelin concurrent changes among brain anatomical structures but limited to studying its predictive value for electrophysiological functional connectivity.

In this study, we use in vivo measures of MT to investigate patterns of correlations in myelination change between gray matter regions. In particular, we aim to study how myelination, a key microstructural feature of brain tissue, changes synchronously among spatially distant regions to form characteristic networks of myelin covariance (*Myelin‐Nets*). Based on the graph theory framework we explore, for the first time, the topological organization of the *Myelin‐Nets* and its modulation by age. Two age groups (Young‐Age, 20–31 years old; Old‐Age, 60–71 years old) were compared to describe age‐related *Myelin‐Nets* topological changes in an elapsed time of 40 years. The continuous aging trajectory of the topology of the *Myelin‐Nets* was also investigated after the fifth decade of life. This approach could be considered as another step for revealing basic principles of gray matter organization, and how these are modified by aging processes.

## 2 MATERIALS AND METHODS

### 2.1 Participants

The dataset included 562 participants of the CoLaus/PsyCoLaus cohort (Firmann et al., 2008; Preisig et al., 2009) and other research studies carried out at the LREN laboratory. The age range was 18–87 years (277 Females). In our first study, a pool of 151 participants were selected to define two age groups: Young‐Age adults: 73 (35 Females), comprising all participants of the dataset from 20 to 31 years old (mean age = 24.56 years) and Old‐Age adults: 78 (40 Females), from 60 to 71 years old (mean age = 65.04 years) (see Figure 3, Results). The age groups were selected with a gap of 40 years to capture different lifespan stages of the myelination processes.

Owing to the reduced number of participants in our cohort between 30 and 47 years old, our second study, aiming to explore the continuous aging trajectories of the *Myelin‐Nets*, was limited to the 48–87 years old age range, which involved a subset of 437 out of the 562 subjects of our original data.

### 2.2 Ethics statement

The study participants gave written informed consent at the time of their enrollment and completed questionnaires approved by the local Ethics Committee. The authors state that they have obtained approval from CHUV and Colaus Data Sharing and Publications Committee for use of the data and confirm that the data was analyzed anonymously.

### 2.3 MRI acquisition and preprocessing

Participants were examined on a 3 T whole‐body MRI system (Magnetom Prisma, Siemens Medical Systems, Germany), using a 64‐channel RF receive head coil and body coil for transmission. On visual inspection study participants showed neither macroscopic brain abnormalities, that is, major atrophy, nor signs of overt vascular pathology (i.e., microbleeds and white matter lesions. Participants with extended atrophy or with white matter hyperintensities (WMH) of grade 2 or more by the Scheltens rating scale (Scheltens et al., 1993) were not included.

The whole‐brain quantitative protocol comprised three multi‐echo 3D fast low angle shot (FLASH) acquisitions with predominantly Magnetization Transfer‐weighted (MTw: TR/α = 24.5 ms/6°), proton density‐weighted (PDw: TR/α = 24.5 ms/6°) and T1‐weighted (24.5 ms/21°) contrast (Helms et al., 2008a, 2008b, 2009). The MTw contrast was achieved by use of a Gaussian‐shaped RF pulse prior to the excitation (4 ms duration, 220° nominal flip angle, 2 kHz frequency offset from water resonance). Multiple gradient echoes were acquired with alternating readout polarity with a minimal echo time TE of 2.34 ms and a time span of 2.34 ms between consecutive echoes; 6/8/8 echoes were acquired for the MTw/PDw/T1w acquisitions to keep the TR value identical for all acquisitions. The image resolution was 1 mm^3^ isotropic, the field of view was 256 × 240 × 176 mm and the matrix size was 256 × 240 × 176. Parallel imaging was used along the phase‐encoding direction (acceleration factor 2 GRAPPA reconstruction (Griswold et al., 2002)), 6/8 partial Fourier was used in the partition direction. The acquisition time was 7 min per contrast. Data were acquired to calculate maps of the radio frequency (RF) transmit field B1+ using a 3‐D echo‐planar imaging (EPI) spin‐echo (SE) and stimulated echo (STE) method (Lutti, Hutton, Finsterbusch, Helms, & Weiskopf, 2010; Lutti et al., 2012) and to correct for effects of RF transmit inhomogeneities on the quantitative maps (Helms et al., 2009; Helms and Dechent, 2009; Weiskopf et al., 2013). The image resolution of the B1‐mapping data was 4 mm^3^. The echo time was 39.06 ms and TR was set to 500 ms. The spin‐echo flip angle was decreased from 230° to 130° in steps of 10° (Lutti et al., 2012). The acquisition time of the B1 mapping data was 3 min. B0‐field mapping data was acquired using a 2‐D double‐echo FLASH sequence to correct for geometric distortions in the 3‐D EPI data as described in Lutti et al. (2010, 2012). The total acquisition time was 27 min.

Calculation of the quantitative Magnetization Transfer (MT) maps from the acquired data was implemented with the Voxel‐Based Quantification toolbox (Draganski et al., 2011) running under SPM12 (Wellcome Trust Centre for Neuroimaging, London, UK; http://www.fil.ion.ucl.ac.uk/spm) and Matlab 7.11 (Mathworks, Sherborn, MA, USA). The MT maps were computed as described in Helms, Dathe, and Dechent (2008a) and Helms and Dechent (2009) using the MTw, PDw, and T1w images with minimal echo time (TE = 2.34 ms) to minimize R2* bias on the MT estimates (Lorio et al., 2016). The amplitude of the MT effect is governed by the duration, power and off‐resonance frequency of the MT saturation pulse. Therefore, the MT effect is most often characterized using semi‐quantitative measures, that is, that depend on the set of acquisition parameters kept constant for all study participants. Note that these MT measures differ from the common Magnetization Transfer Ratio (MTR), by accounting for local T1 relaxation and flip angle inhomogenity effects, resulting in enhanced robustness and sensitivity to myelin concentration (Helms et al., 2008b).

### 2.4 Construction of the myelin data matrix

**Figure 1:**
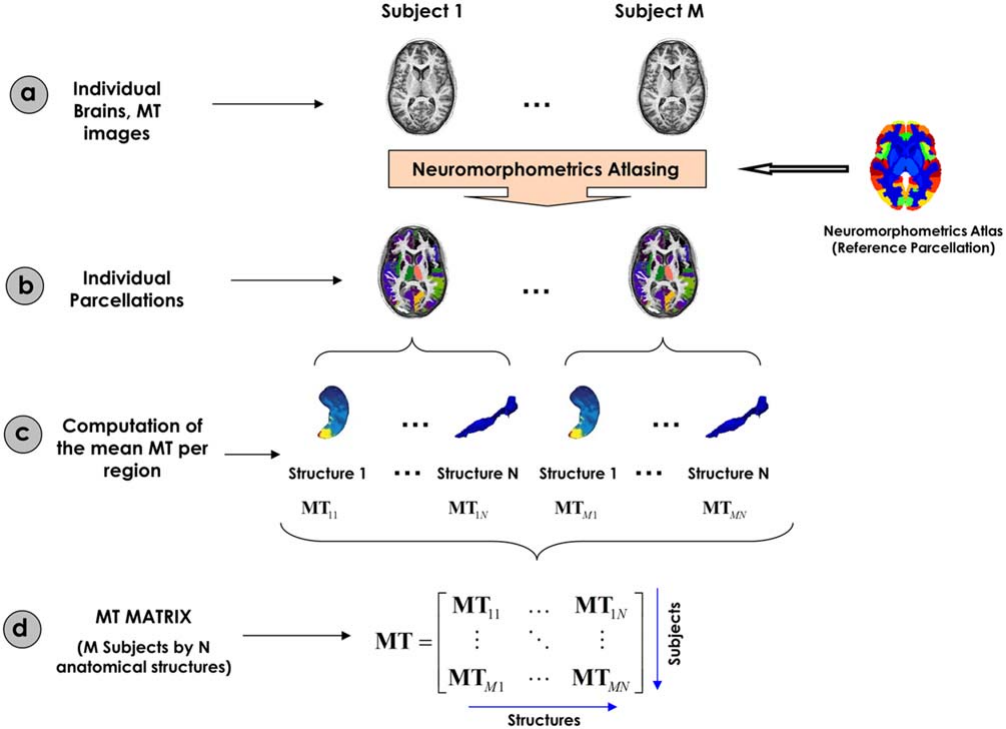
Flowchart of the MT matrix construction. (a) Representation of the individual MT maps for all subjects. (b) Neuromorphometrics Atlasing processing for the parcellation of the individual MT maps. (c) Mean MT values are computed for all anatomical structures. (d) The local MT values were organized in a “Myelin Data” matrix [Color figure can be viewed at http://wileyonlinelibrary.com]

Figure 1 shows the flowchart representing the construction of the matrix containing the local MT estimates for all subjects (Myelin Data matrix). The MT maps (step 1) were automatically parcellated into 114 gray matter regions based on Neuromorphometrics atlas (our main gray matter parcellation containing cortical and subcortical structures) using the Neuromorphometrics toolbox (unpublished, John Ashburner personal communication; see Supporting Information, Table SI for the full list of the structures of the Neuromorphometrics atlas). The Myelin data matrix was *M* × *N*, where “*M*” represents the number of subjects and “*N*,” the number of anatomical structures.

In short, the Neuromorphometrics atlasing methodology (Figure 1, step 2) consists of two main steps. First, each individual MT image is segmented into three different brain tissue classes (cerebral spinal fluid, gray matter and white matter) using the “Segment” (unified segmentation) tool in SPM12, which includes a registration to MNI (Montreal Neurological Institute) space. Second, the probabilistic atlas of each of the 114 anatomical structures of the Neuromorphometrics atlas (see further) are spatially registered with the extracted gray and white matter tissue maps using the “Shoot” tool in SPM12, based on a nonlinear advanced registration algorithm (Ashburner and Friston, 2011). Rules of probability are used to properly combine the previous images to ultimately obtain a probabilistic label map for each brain structure.

At every gray matter voxel (in subject space), the probability of belonging to a specific anatomical structure is provided. From above, a maximum probability label maps are calculated at all gray matter voxels (in subject space) which are labeled according to the structure of maximum probability. Finally (Figure 1, steps 3 and 4), mean MT values are calculated across voxels belonging to each structure label and are used as a proxy for local measures of myelin content. The “Neuromorphometrics” probabilistic and maximum probability tissue labels were derived from the “MICCAI 2012 Grand Challenge and Workshop on Multi‐Atlas Labeling” (https://masi.vuse.vanderbilt.edu/workshop2012/index.php/Challenge_Details). These data were released under the Creative Commons Attribution‐Non‐Commercial (CC BY‐NC) with no end date. The anatomical T1‐weigthed MRI scans originate from the OASIS project (http://www.oasis-brains.org/) and the labeled data were provided by Neuromorphometrics, Inc. (http://Neuromorphometrics.com/) under academic subscription.

### 2.5 Construction of the myelin correlation matrix

**Figure 2:**
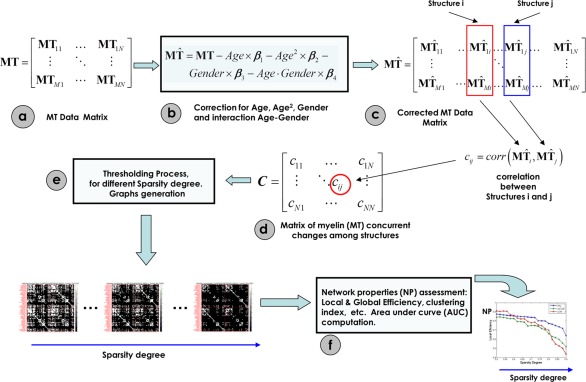
Steps for the assessment of the Myelin correlation matrices (*Myelin Nets*). (a) MT data matrix. (b) The MT original data matrix is substituted by residuals of the linear regression represented in (c). (c) Effects of age, age^2^, gender, and age–gender interaction were regressed out. (d) Correlation matrix representing the myelin concurrent changes among all pairs of anatomical structures. (e) Thresholding process at different sparsity degrees to generate binary graphs. (f) Assessment of the network properties for all binary graphs obtained in (e) [Color figure can be viewed at http://wileyonlinelibrary.com]

The process of constructing the myelin correlation matrix is summarized in Figure 2. For each age group, a linear regression was performed on the regional MT estimates to remove the effects of age, age^2^, gender, and age–gender interaction (Figure 2, steps b and c). The residuals of this regression then replaced the raw values in the myelin data matrix. This detrending step was implemented to remove the effects of the mean MT values—and their dependence on age—likely to bias the covariance estimates by overestimating the Pearson correlation. Only fluctuations around the mean MT ageing trajectories are of interest in this analysis. The correlation between global and local MT was statistically the same for both groups (*p* = .85) and different sliding windows (*p* > .05) in the aging trajectory study (Supporting Information, Study 1). Therefore, the global MT was not regressed out as confounding variable. Global MT effect subtraction in principle may highlight interregional differences but at the same time will affect genuine covariance patterns (and topological network attributes) without the possibility of estimating the introduced bias (Borchardt et al., 2016; Fox, Zhang, Snyder, & Raichle, 2009; Murphy, Birn, Handwerker, Jones, & Bandettini, 2009; Murphy and Fox, 2017; Schwarz and McGonigle, 2011).

We defined a connection as the statistical association in MT values (a surrogate measure of myelin) between each pair of brain regions for a parcellation scheme of 114 anatomical structures. The statistical similarity or synchronized co‐variations in myelination between two regions was measured by computing the Pearson's correlation coefficient across subjects (Figure 2, steps c and d) (He et al., 2007). Hence, the interregional Pearson's correlation matrix *C* (*N* × *N*, *N* (=114) being the number of gray matter regions)—or *Myelin correlation matrix—*contains elements *C_ij_* that are the value of the Pearson's correlation between regions *i* and *j*. Self‐connections were excluded, implying zeros in the diagonal of this symmetric matrix.

It is important to point out that a partial correlation analysis could not be used in our case because the sample size was not large enough for a robust estimation of this measure (i.e., the number of structures in the Neuromorphometrics parcellation is higher than the number of subjects for each group).

In the next step, we obtained for each age group Nboot = 2000 bootstrap samples of the connectivity matrix by selecting a random subset of subjects with replacement using the classical bootstrapping procedure described in (Efron and Tibshirani, 1994). This algorithm guarantees independent samples. Bootstrapping allows the computation of empirical distributions of the connectivity matrices and of the topological network properties (i.e., mean, standard deviation, etc.) that, with a sufficient number of data points, reflect the true underlying distributions. This could not be obtained with a permutation resampling approach. In addition, and very, important the bootstrapped connectivity matrices allowed us to estimate the significance of changes in network properties between age groups (Young‐Age and Old‐Age) taking into account the variability of these properties within each group. The connectivity matrices obtained from the 2000 bootstraps were thresholded to create sparse binary graphs (Figure 2, step e). Rather than restricting our analysis to a binarized graph obtained by applying a single threshold value, we explored the Network Properties of the graphs over a range of sparsity degrees varied from 0.5 to 0.9 in steps of 0.02 (a sparsity degree of 0.9 means that 90% of the connectivity matrix is discarded, keeping only the highest 10% of the connectivity values) (Figure 2, step f) (Bassett, Nelson, Mueller, Camchong, & Lim, 2012; Ginestet, Nichols, Bullmore, & Simmons, 2011; Sanabria‐Diaz et al., 2010). This range of sparsity degree was chosen to allow for all network properties to be accurately estimated and the number of spurious edges in each network minimized as indicated in previous studies (Achard and Bullmore, 2007; He et al., 2007). Corresponding threshold values *R_k_* were calculated for each of the 2000 correlation matrices so that their elements *C_ij_* were set to 1 when |*C_ij_*| > *R_k_* and 0 otherwise. This procedure normalizes the networks to have the same number of nodes and edges, enabling the examination of the relative network properties obtained for each group.

### 2.6 Graph analysis to characterize the “networks of myelin covariance” (Myelin‐Nets)

A great number of natural systems can be represented by complex networks. Graph Theory is usually considered an attractive model for the mathematical treatment of such networks, including those representing brain connectivity (Sporns, 2011). In general, a complex network can be represented as a graph *G* = [*N*,*K*], where the nodes *N* are the components of this system and the edges (*K*), the relations or connections between them (Boccaletti, Latora, Moreno, Chavez, & Hwang, 2006). In our specific case, the nodes are the anatomical regions obtained from the brain parcellation and the edges are the co‐variations (correlation) in myelination across subjects between pairs of these brain regions.

It is important to note here that this is a mathematically derived network, whose connections do not explicitly reflect anatomical or physiological mechanisms in the brain. However, because these “Networks of myelin covariance” (*Myelin‐Nets*) are based on anatomical data, they can be taken as possible biomarkers of underlying biophysical mechanisms. These *Myelin‐Nets* are unweighted because all edges are assumed to indicate relations of equivalent strength between nodes, and undirected, simply summarizing symmetric relations (such as correlations) between nodes.

We used graph theory to study the myelin co‐variation networks in the Young‐Age and Old‐Age groups. This mathematical treatment allows us to characterize the age‐related changes of global and local phenomena observed when myelination in any structure fluctuates concurrently with myelination in its neighborhoods and other distant brain regions of the network. In other words, graph theory gives us a framework to explore the *Myelin‐Nets* architecture and how efficiently the information of myelin fluctuations is “exchanged” over the network (in terms of the graph theory). Importantly, these networks cannot be interpreted in terms of temporal causality. First, because myelination co‐variations are assessed across subjects and second, because we are using Pearson correlations, measures that do not provide directional/causal information on the interactions between pairs of nodes.

In particular, we analyzed the following global network attributes: cluster index, local and global efficiency, and characteristic path length. To describe the nodal properties of the network we computed the betweenness centrality attribute that allowed us to identify the network hubs. Additionally we carried out a “Targeted Attack” study to evaluate the resilience of the *Myelin‐Nets* when the most important regions (hubs) are virtually attacked. In the following, these measures will be defined with the traditional interpretation of general networks. However, their usefulness as relevant descriptors of anatomical brain states will become apparent in the next sections.

### 2.7 Clustering index (*C*)

The clustering index $C_{i}$ of a node “*i*” is defined as the number of existing connections between the node's neighbors divided by all their possible connections. It is a measure of the inherent tendency of nodes to cluster into strictly connected neighborhoods (Watts and Strogatz, 1998). Nodes are considered neighbors when a connection between them exists, which is not reduced to a physical neighborhood concept. The clustering index for the whole graph *G* is defined as the average clustering around each node:

(1) $$C=\frac{1}{N}\sum_{i\in G}C_{i}$$

represents the number of nodes. Clearly, 0 < *C* < 1; and *C* = 1 if and only if the network is fully connected; that is, each node is connected to all other nodes.

### 2.8 Characteristic path length (*L*)

The characteristic path length *L* of the graph G is the smallest number of connections required to connect one node to another, averaged over all pairs of nodes. It is a measure of the typical separation between two nodes (structures) *i* and *j* (∀i,j∈N), and it is defined as the mean of geodesic lengths $d_{ij}$ over all pairs of nodes.

(2) $$L=\frac{1}{N\left(N-1\right)}\sum_{\begin{array}{l} i,j\in G\\ i\neq j \end{array}}d_{ij}$$

In the unweighted network context, the geodesic length $d_{ij}$ is defined as the number of edges along the shortest path connecting nodes *i* and *j* (Boccaletti et al., 2006; Watts, 1999; Watts and Strogatz, 1998).

### 2.9 Network efficiency

The concept of efficiency has also been expressed in terms of information flow (Latora and Marchiori, 2001). That is, small world networks are very efficient in terms of global and local communication and they are defined to have high global $E_{\text{glob}}$ and local $E_{\text{loc}}$ efficiency. The global $E_{\text{glob}}$ of a graph G is expressed as:

(3) $$E_{\text{glob}}=\frac{1}{N\left(N-1\right)}\sum_{\begin{array}{l} i,j\in G\\ i\neq j \end{array}}\frac{1}{d_{ij}}$$

This measure reflects how efficiently the information can be exchanged over the network, considering a parallel system in which each node sends information concurrently along the network. On the other hand, the $E_{\text{glob}}$of *G* is defined as the average efficiency of the local subgraphs:

(4) $$E_{\text{loc}}=\frac{1}{N}\sum_{i\in G}E_{\text{glob}}\left(G_{i}\right)$$

where $G_{i}$ is the subgraph of the neighbors of “*i*.” This measure reveals how much the system is fault tolerant, showing how efficient the communication is among the first neighbors of *i* when it is removed (Latora and Marchiori, 2001). As above, nodes are considered neighbors when a connection between them exists, which is not reduced to a physical neighborhood concept.

### 2.10 Nodal centrality: Normalized betweenness centrality (NBC)

The “betweenness centrality” *B_i_* of a node *i* is defined as the number of shortest paths between any two nodes that run through node *i* (Freeman, 1977). We measured the normalized betweenness centrality as *b_i_* = *B_i_*/<B>, where <B> was the average betweenness of the network. *b_i_* is a global centrality measure that captures the influence of a node over information flow between other nodes in the network. In our case, betweenness centrality *b_i_* could be used to reflect the effects of aging on the global roles of regions in the *Myelin‐Nets*. Hubs were selected as those with *b_i_* superior to 1.5 similarly to what has been proposed in previous investigations (He, Chen, & Evans, 2008; Melie‐García, Sanabria‐Diaz, & Sánchez‐Catasús, 2013; Yao et al., 2010; Zhu et al., 2012).

### 2.11 Methodology for studying differences in the myelination correlation across brain lobes

To investigate differences in myelination correlation (interconnectivity) between age groups across brain lobes we used the anatomical subdivision of the brain in lobes proposed by Tzourio‐Mazoyer et al. (2002). We assessed the intra lobe myelination connectivity as the mean of the absolute correlation coefficient values (first converting the absolute correlation coefficient values to z using Fisher's r‐to‐z transformation, taking the mean and transforming back to correlation through the inverse Fisher transformation) among intralobe structures in the limbic, frontal, parietal, occipital, temporal lobes, insula, and subcortical nuclei for each group and all bootstrap samples of the correlation matrices. To test differences between groups, we used the statistical procedure described below.

### 2.12 Statistical methods to study aging modulation of Myelin‐Nets properties

Network properties (NP) of the myelination correlation matrices were computed for each sparsity degree values and different bootstrap samples in each age group. Thus, we had a set of Nboot = 2000 NP curves for each network property showing the change in NP with the sparsity degree. The area under the curve was computed for each network attribute to contrast the global behavior of these attributes (He et al., 2009; Wu et al., 2011b). It is worth noting that the monotonic changes of the NP curves with the sparsity degree make the area under the curve a suitable descriptor of the global performance of the networks.

We followed three main steps to examine differences in network properties between groups: (a) construction of the empirical bootstrapped distribution of differences by subtracting the corresponding bootstrap samples between groups; (b) definition of the statistical significance level: a 95% confidence interval (CI) (biased corrected percentile bootstrap CI) (Efron, 1982) of the empirical differences distribution is estimated; (c) hypothesis testing: a significant difference between groups is accepted if CI does not contain zero, no significant difference is considered otherwise.

### 2.13 Methodology to study robustness of the Myelin‐Nets: Targeted attack analysis

We calculated a surrogate measure of the resilience of the Myelin‐Nets against targeted attack. In a simulated targeted attack study, network hubs are removed one by one in order of betweenness centrality (NBC). Each time a node was removed from the network, the size of the largest connected component was recomputed. We defined the robustness parameter as the area under the curve showing the size of the relative largest connected component versus the number of nodes removed (Achard, Salvador, Whitcher, Suckling, & Bullmore, 2006). Robust networks retain large connected components even when several nodes have been knocked out, as represented by a large area under targeted attack curve. As before, we repeated this procedure for each Nboot = 2000 bootstrapped connectivity matrices in the 21 sparsity degree points. The same statistical procedure used for evaluating aging effect of *Myelin‐Nets* properties was applied to explore network robustness differences between groups.

### 2.14 Age modulation of the Myelin‐Nets global network properties: influence of the gray matter parcellation

There is empirical evidence that topological properties of brain networks depend on the gray matter parcellation used (Zalesky et al., 2010). In order to study the effect of grey matter parcellation on the *Myelin‐Nets* and their modulation with age, we selected, in addition to the Neuromorphometrics atlas, three alternative atlases of diverse nature, different number and distributions of anatomical structures. These atlases were (a) AAL atlas with *N* = 90 structures (Tzourio‐Mazoyer et al., 2002); (b) Brainnetome Atlas (Fan et al., 2016) with *N* = 246 structures, a cross‐validated atlas containing information on both anatomical and functional connections, and (c) Gordon atlas (Gordon et al., 2016) with *N* = 333 cortical regions based on the homogeneity of resting‐state functional connectivity patterns. The *Myelin‐Nets* and its topological attributes were assessed for each of these atlases as described in the previous sections.

### 2.15 Methodology for studying the aging trajectory of the Myelin‐Net's global network attributes

To uncover dynamical properties of the *Myelin‐Nets* topological organization with age, we proposed a continuous aging trajectory analysis based on a sliding window approach. This methodology has been used in two forms to study networks of anatomical covariance: (a) using a weighted contribution of the data points in the windows to the correlation coefficient at the age window centers (Zalesky et al., 2015); (b) using a fixed number of data points (subjects) per window, the overlap between contiguous windows being selected heuristically (Vasa et al., 2017). Here, we kept the number of subjects per window constant, equal to the number of subjects of the Young‐Age group (73), to keep the correlation matrices and their topological features across windows unbiased by the number of data points. For each slid, the youngest subject of the current window was replaced with the nearest older participant (i.e., step size = 1: two contiguous windows only differed by one subject). This process was repeated iteratively across the age range = 48–75 years old (Section 2.1). For each sliding‐window, the *Myelin‐Nets* and their topological attributes were calculated using the methodology described in *Construction of the Myelin correlation matrix* section without bootstrapping. The sliding window “age” was defined as the median age of the participants in each window. The minimum “age” difference between contiguous windows was 0.01 (∼4 days) and the maximum was 0.6 (∼half a year). The maximum age difference between the youngest and oldest subjects within a window was 9.16 years (around the “window age” = 74 years old), the minimum 3.29 years and the mean 5.3 years.

We focused our attention on the main global network attributes of the *Myelin‐Nets*: clustering index, characteristic path length, local and global efficiency, global connectivity, and the connectivity strength between homologous regions. Additionally a study of the aging trajectory of the normalized betweenness centrality (NBC), as nodal network property, was performed.

The aging trajectory of the *Myelin‐Nets* attributes were fitted as a function of the “window age” using a polynomial model. The order of the polynomial fitting was determined by the Akaike's Information Criterion (AIC) (Bozdogan, 1987). The statistical significance of the polynomial coefficients was assessed through Student's *t* test in the linear regression model.

## 3 RESULTS

### 3.1 Global gray matter myelination changes with age

**Figure 3:**
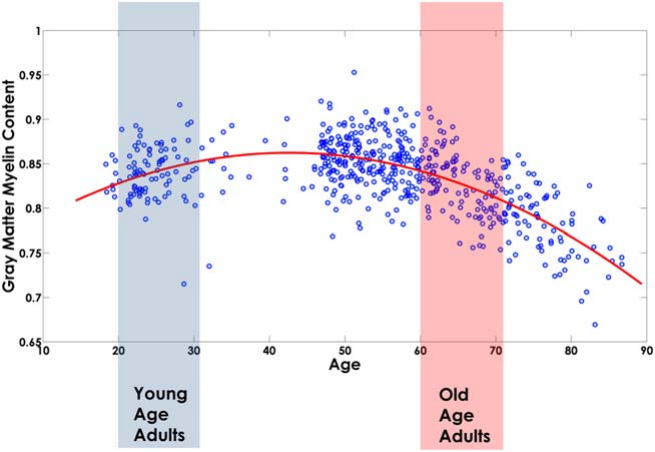
Global gray matter MT values versus age for the 562 subjects of the cohort. The MT values (blue scatter plot) followed an inverted‐U shape trajectory that was fitted with a second‐order polynomial model (red plot). The blue and red boxes highlight the age range of the Young and Old Age categories that were used in the covariance analysis [Color figure can be viewed at http://wileyonlinelibrary.com]

Before detrending for age, visualization of the full 562 dataset showed that the global myelination in gray matter followed an inverted‐U shape trajectory with age (Figure 3).

This motivated the use of a second‐order polynomial model to fit the age dependence of the MT data for the detrending (using “polyfit.m” subroutine implemented in MATLAB 2015a), with significant coefficients (*p* < .05). Aging was found to have a heterogeneous effect on myelination across gray matter regions (Supporting Information, Figures S1–S4). The peak in myelination was found to take place between 40 and 50 years of age for most of brain anatomical regions (Supporting Information, Figure S2). The modeled age‐dependence of the regional MT values was detrended from the original values before calculation of the correlation matrices.

### 3.2 Aging modulates the correlation strength between homologous regions but not the global myelin correlation strength

**Figure 4:**
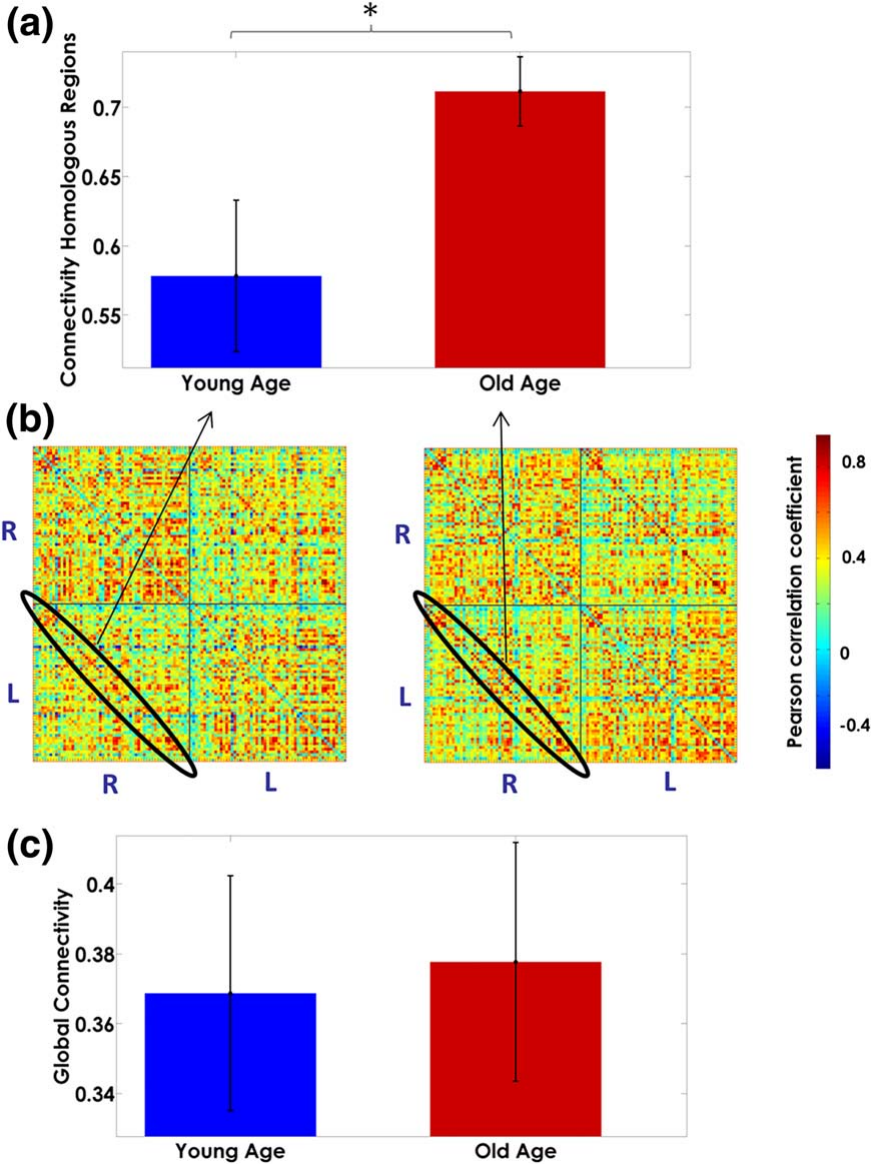
Age modulation of the correlation strength between homologous regions (Panel a). Panel b depicts the correlation matrices for each group. The global myelination correlation strength in both Young‐Age and Old‐Age groups is represented in panel c. The bar's height represents the mean magnitude of the correlation, and the error bars represent their standard deviations. In panel b, the “R–R” and “L–L” quadrants represent the intrahemispheric myelin correlations in the right and left hemispheres, respectively. The “R–L” and “L–R” quadrants depict the interhemispheric interactions. The diagonal of the “L–R” quadrant, highlighted in black shows the correlations in myelination between homologous structures across hemispheres. The asterisk denotes significant differences between groups [Color figure can be viewed at http://wileyonlinelibrary.com]

Figure 4 (panel b) shows the MT correlation matrices for each age group. We found a significant effect of age on the mean MT correlations between homologous regions (Figure 4, Panel a, b), the Old‐Age group showing significantly (*p* < .05) higher correlations than the Young‐Age group. However no statistical difference in global connectivity was found between groups (Figure 4, panel C). The results of the statistical analysis including confidence intervals can be found in Table SIV, Supporting Information.

### 3.3 Aging modulates the correlations in myelination in brain lobes

**Figure 5:**
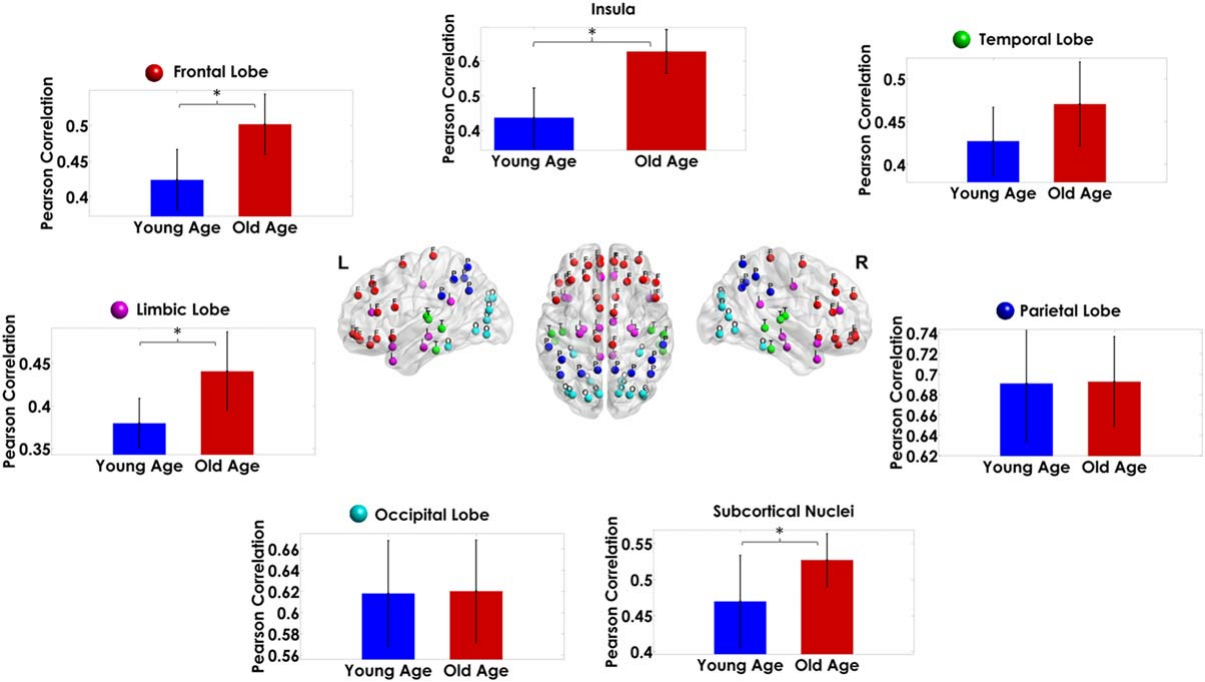
Modulation of the strength in myelination correlation by age within the brain lobes and the subcortical nuclei. The height of the bars represents the mean magnitude of the correlations and the error bars their standard deviation. The asterisks denote significant differences between groups. For reference, we show at the center of the figure the distribution of the nodes in different colors for each lobe [Color figure can be viewed at http://wileyonlinelibrary.com]

As shown in Figure 5, the correlation in myelination within brain lobes (pulled from both hemispheres) is differently modulated by age. The Old‐Age group showed higher intra‐lobe correlation strength than the Young‐Age group except for the temporal, parietal, and occipital lobes, where no significant differences were found (the detailed statistical results can be found in Supporting Information, Table SII).

### 3.4 Most connected structures in Myelin‐Nets

**Figure 6:**
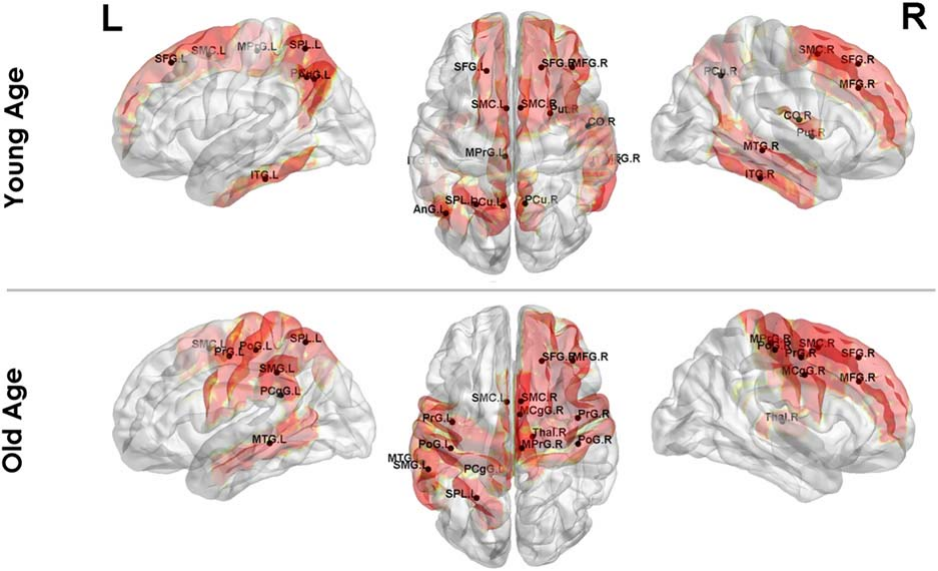
Top 15 regions with the largest myelin covariance (most connected) in the *Myelin‐Nets* for the Young and Old Age Groups [Color figure can be viewed at http://wileyonlinelibrary.com]

In a more detailed study of the correlations in myelination, we found a set of structures where the variations in myelination were particularly strongly correlated with myelination changes in other gray matter regions. Figure 6 shows the 15 structures with the strongest mean inter‐regional covariations in each age group (the cortical surface plots were created using the BrainNet Viewer package (http://www.nitrc.org/projects/bnv) (Xia, Wang, & He, 2013)). Table 1 provides the full list of these structures, highlighting in bold those that are common for both age groups.

**Table 1:** The 15 regions with the highest inter‐regional correlations in myelination for the Young and Old Age Groups

Young Age Group		Old Age Group	
Structure name	Corr. value	Structure name	Corr. value
Precuneus (PCu.L)	0.492	Precentral gyrus (PrG.R)	0.493
Middle temporal gyrus (MTG.R)	0.472	Supramarginal gyrus (SMG.L)	0.470
Precentral gyrus medial segment (MPrG.L)	0.470	**Middle frontal gyrus (MFG.R)**	**0.467**
Central operculum (CO.R)	0.458	**Superior parietal lobule (SPL.L)**	**0.464**
Inferior temporal gyrus (ITG.R)	0.447	Postcentral gyrus (PoG.L)	0.462
**Superior parietal lobule (SPL.L)**	**0.446**	Postcentral gyrus (PoG.R)	0.456
Precuneus (PCu.R)	0.446	Precentral gyrus medial segment (MPrG.R)	0.452
**Supplementary motor cortex (SMC.L)**	**0.444**	Middle cingulate gyrus (MCgG.R)	0.452
Angular gyrus (AnG.L)	0.437	Posterior cingulate gyrus (PCgG.L)	0.450
Superior frontal gyrus (SFG.R)	0.437	**Supplementary motor cortex (SMC.R)**	**0.447**
**Supplementary motor cortex (SMC.R)**	**0.436**	**Superior frontal gyrus (SFG.R)**	**0.447**
**Superior frontal gyrus (SFG.L)**	**0.432**	Middle temporal gyrus (MTG.L)	0.444
**Middle frontal gyrus (MFG.R)**	**0.430**	Precentral gyrus (PrG.L)	0.442
Putamen (Put.R)	0.428	Thalamus Proper (Thal.R)	0.440
Inferior temporal gyrus (ITG.L)	0.424	**Supplementary motor cortex (SMC.L)**	**0.440**

The structures present in both groups are highlighted in bold.

The most connected regions were the Precuneus Left (PCu.L) and Precentral Gyrus (PrG.R) for the Young and Old Age groups respectively. The structures common to both groups were left superior parietal lobule (SPL.L), left and right supplementary motor cortex (SMC.L, SMC.R), right superior frontal gyrus (SFG.R), and the middle frontal gyrus (MFG.R). Interestingly, the most connected region in each group is not present in the other.

### 3.5 Effects of age on the Myelin‐Nets hubs

**Figure 7:**
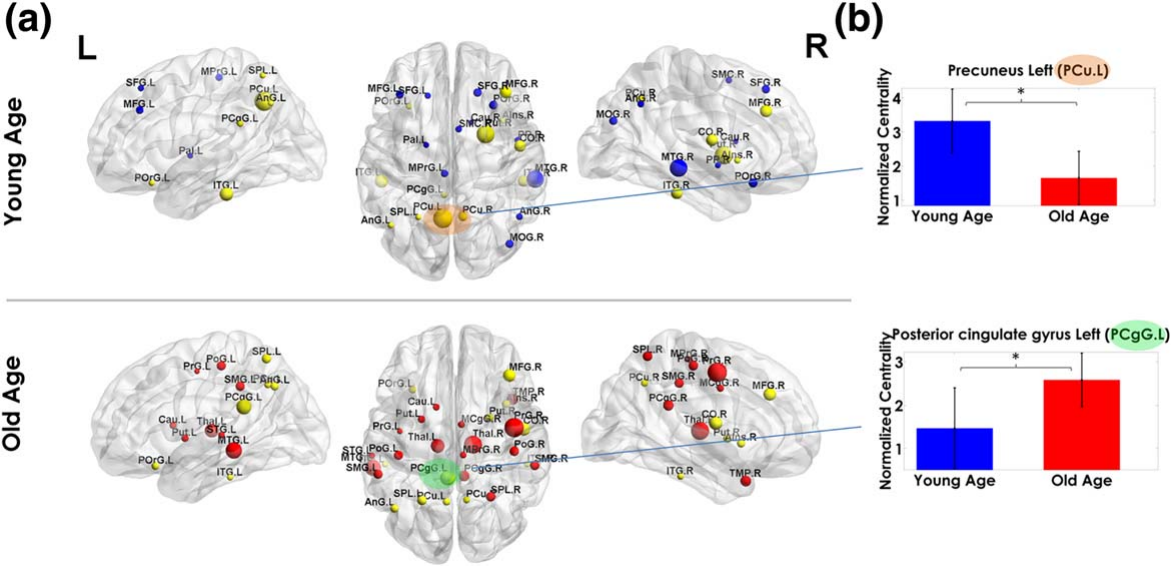
Hub regions in Young‐Age and Old‐Age groups (Panel a). Panel b shows the modulation of the normalized betweenness centrality (NBC) by age in the Precuneus left (PCu.L, orange shaded) and Left Posterior cingulate gyrus (PCgG.L, green shaded): the regions with the highest NBC Young and Old Age groups, respectively. The sphere diameter denotes the NBC values. Spheres in yellow are hubs common in both groups. The blue and red hubs are those unique to the Young and Old age groups, respectively. In panel b, the bar heights represent the mean magnitude of the NBCs and the error bars represent their standard deviation. The asterisks denote significant differences between groups [Color figure can be viewed at http://wileyonlinelibrary.com]

We also studied the effects of age on the hubs of the *Myelin‐Nets*, defined as having a Normalized Betweenness Centrality (NBC, a quantitative measure of the importance of structures in the *Myelin‐Nets*) over 1.5 (Figure 7, panel A).

The full list of hub structures can be found in Table 2. Left precuneus (PCu.L) and left middle temporal gyrus (MTG.L) were the structures with the highest NBC for the Young‐Age and Old‐Age groups, respectively. Twelve hubs were common to both age groups (yellow spheres in Figure 7, panel A). Among the hub structures, we examined the age modulation of the NBC for the regions with the highest NBC for each group (Precuneus Left (PCu.L) and left posterior cingulate gyrus (PCgG.L) for Young‐Age and Old‐Age groups, respectively (Figure 7, panel b). The PCu.L showed significantly higher NBC in Young‐Age than Old‐Age. On the contrary, NBC of the PCgG.L was significantly higher in the Old‐Age group (the detailed statistical results can be found in Supporting Information, Table SIII). The aging trajectory of both PCu.L and PCgG.L can be found in Supporting Information, Figure S3.

**Table 2:** List of hub regions for the Young and Old age groups, defined as the nodes with Normalized Betweenness Centrality above 1.5

Young Age Group		Old Age Group	
Structure name	NBC	Structure name	NBC
**Precuneus (PCu.L)**	**3.8849**	Middle temporal gyrus (MTG.L)	3.7686
**Putamen (Put.R)**	**3.425**	Precentral gyrus (PrG.R)	3.7563
Middle temporal gyrus (MTG.R)	3.2859	Thalamus Proper (Thal.R)	3.5141
**Inferior temporal gyrus (ITG.L)**	**2.656**	**Central operculum (CO.R)**	**3.0556**
**Middle frontal gyrus (MFG.R)**	**2.5353**	Thalamus Proper (Thal.L)	3.071
**Central operculum (CO.R)**	**2.5039**	**Posterior cingulate gyrus (PCgG.L)**	**2.8282**
**Precuneus (PCu.R)**	**2.3277**	**Middle frontal gyrus (MFG.R)**	2.8084
**Inferior temporal gyrus (ITG.R)**	**2.2482**	Temporal pole (TMP.R)	2.4644
Superior frontal gyrus (SFG.R)	2.1854	Supramarginal gyrus (SMG.L)	2.458
Posterior orbital gyrus (POrG.R)	2.1587	Supramarginal gyrus (SMG.R)	2.2991
Precentral gyrus medial segment (MPrG.L)	2.1213	**Superior parietal lobule (SPL.L)**	**2.2354**
**Anterior insula (AIns.R)**	**1.9716**	Posterior cingulate gyrus (PCgG.R)	2.1277
Middle occipital gyrus (MOG.R)	1.939	Postcentral gyrus (PoG.R)	2.0551
Supplementary motor cortex (SMC.R)	1.8457	**Putamen (Put.R)**	**1.9975**
**Superior parietal lobule (SPL.L)**	**1.7905**	**Posterior orbital gyrus (POrG.L)**	**1.9523**
**Angular gyrus (AnG.L)**	**1.762**	**Anterior insula (AIns.R)**	**1.929**
Angular gyrus (AnG.R)	1.7384	Putamen (Put.L)	1.8917
Superior frontal gyrus (SFG.L)	1.6695	**Angular gyrus (AnG.L)**	**1.8883**
Planum polare (PP.R)	1.6546	Superior parietal lobule (SPL.R)	1.8642
Middle frontal gyrus (MFG.L)	1.5893	**Inferior temporal gyrus (ITG.L)**	**1.7996**
Caudate (Cau.R)	1.5847	Caudate (Cau.L)	1.7878
**Posterior orbital gyrus (POrG.L)**	**1.5642**	Postcentral gyrus (PoG.L)	1.7645
Supplementary motor cortex (SMC.L)	1.5616	Middle cingulate gyrus (MCgG.R)	1.7474
**Posterior cingulate gyrus (PCgG.L)**	**1.5565**	**Precuneus (PCu.R)**	**1.6615**
Pallidum (Pal.L)	1.5257	**Precuneus (PCu.L)**	**1.6509**
Entorhinal area (Ent.L)	1.5054	**Inferior temporal gyrus (ITG.R)**	**1.6285**
		Precentral gyrus (PrG.L)	1.5645
		Superior temporal gyrus (STG.L)	1.5463

The structures common to both groups are highlighted in bold font.

### 3.6 Effects of age on the properties of the Myelin‐Nets

**Figure 8:**
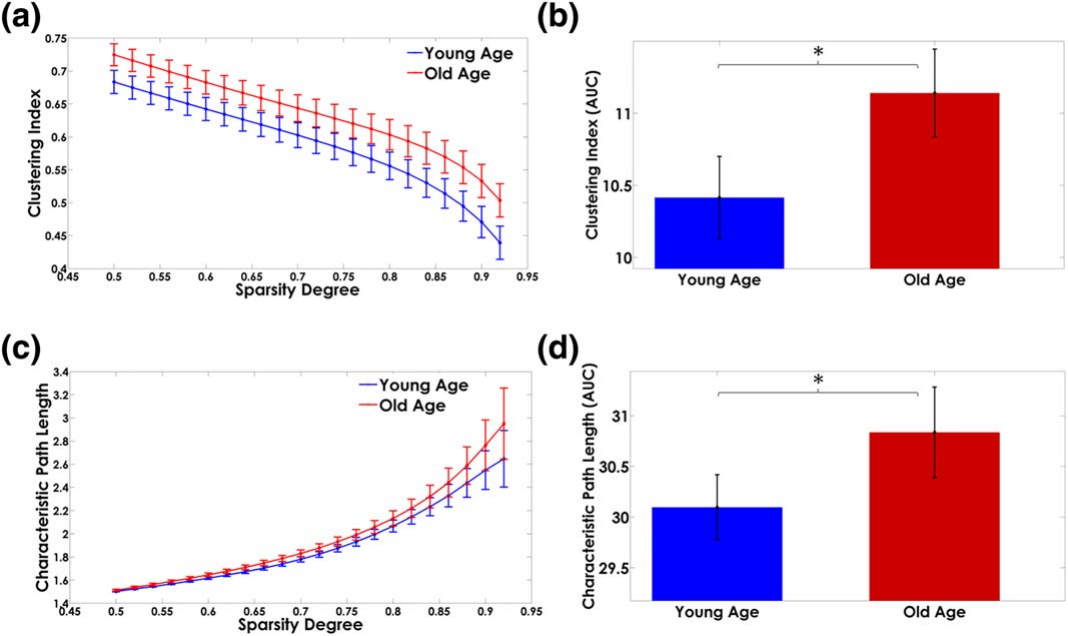
Panels (a) and (c) show the network attributes trajectory for different sparsity degrees. The area under the curves (AUC) of the *Myelin‐Net's* global properties are represented in panels (b) and (d). The bar heights represent the mean of the network properties and the error bars are their standard deviation. The asterisks denote significant differences between groups (*p* < .05) [Color figure can be viewed at http://wileyonlinelibrary.com]

Significant differences in characteristic path length, clustering index, local, and global efficiency were observed between groups (Figure 8).

**Figure 9:**
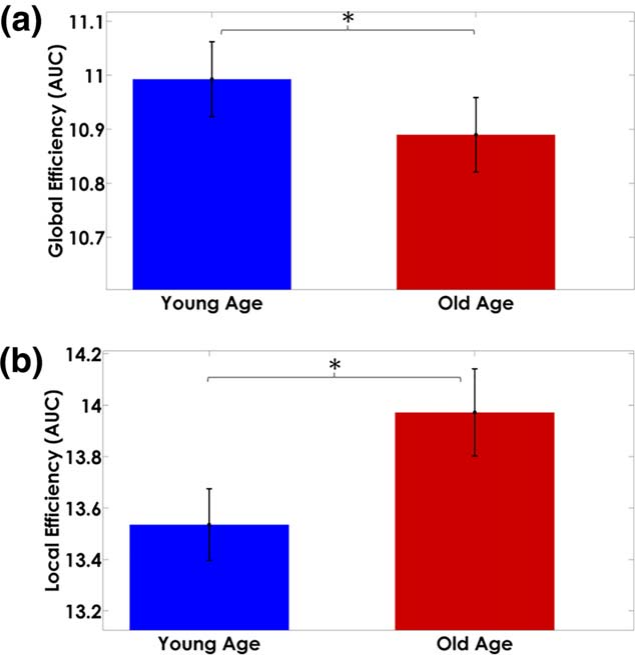
Area under the curve measures of *Myelin‐Net* (a) global and (b) local efficiencies. The bar heights represent the mean of the network properties and the error bars are their standard deviation. The Young‐Age group showed higher global efficiency (*p* < .05) and the Old‐Age higher local efficiency (*p* < .05) [Color figure can be viewed at http://wileyonlinelibrary.com]

The Old‐Age group showed the largest area under the curve (AUC) values for the characteristic path length, clustering index (Figure 8), and local efficiency (Figure 9, panel b).

In contrast, the Young‐Age group depicted higher global efficiency (Figure 9, panel a). Details of the results of the statistical tests can be found in Supporting Information, Table SIV.

### 3.7 Effects of age on the resilience of the myelin networks to targeted attack

**Figure 10:**
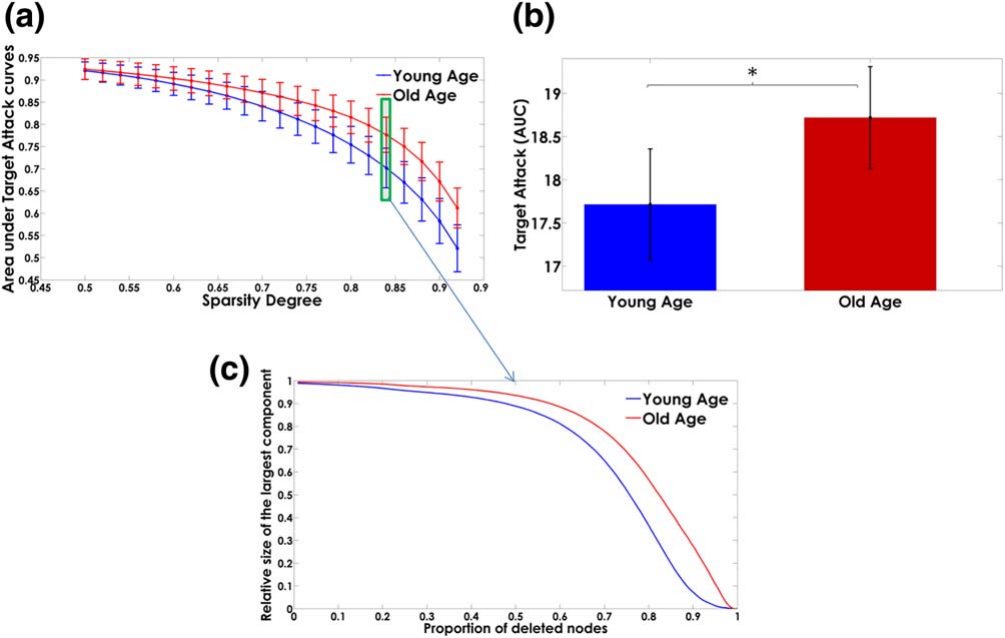
Panel (a) shows the dependence of the “area under targeted attack” on the sparsity degree. The error bars represent the standard deviation over the bootstrap samples. The areas under the “targeted attack” curves are represented in panel (b). The bar heights represent the mean values for the Young‐Age and Old‐Age groups and the error bars the standard deviations. The asterisks denote significant differences between groups. The Old‐Age group showed higher resilience after “simulated attacks” of the *Myelin‐Net* hubs (*p* < .05). Panel (c) represents the trajectories of the relative size of the largest components as the principal nodes are “deleted” (“attacked”) for the sparsity degree highlighted in panel a (green box) [Color figure can be viewed at http://wileyonlinelibrary.com]

In the “Targeted Attack” study, we found that the resilience to virtual damage of the principal structures in the *Myelin‐Nets* increases with age: the Old‐Age group showed higher resilience after “simulated attacks” to the *Myelin‐Net* hubs (Figure 10).

In contrast, the Young‐Age group was more vulnerable than the Old‐Age group. The relative size of its largest component was severely degraded by targeted attack. The difference between groups was statistically significant. Details of the statistical results can be found in Supporting Information, Table SIV.

### 3.8 Aging trajectory of the Myelin‐Net's global network attributes

**Figure 11:**
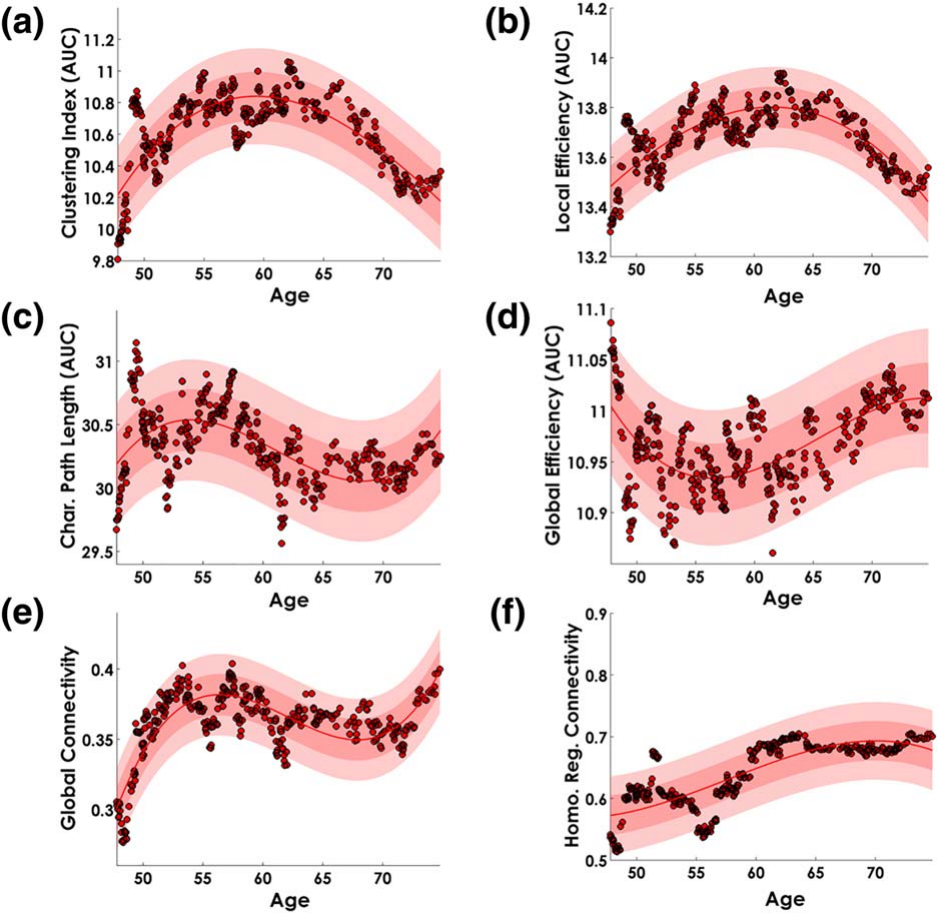
Age trajectory of the MyelinNets Global Network properties. The *Myelin‐Nets* segregation topological measures are represented in panel (a). clustering index, and panel (b), local efficiency. The integration topological measures: characteristic path length and global efficiency are represented in panels (c) and (d), respectively. Panel (e) shows the global connectivity and panel (f), the connectivity strength between homologous regions. The continuous line in red represents the polynomial fitted function. The light red shaded area symbolizes the confidence interval of the polynomial fitted function, and the dark shaded area the standard deviation of the error in predicting a future observation. Dots in red and black represent the topological network property values for each “age”—taken as the median age of the participants in the window. In all cases the best fitted polynomial order, based on the AIC criterion, was *n* = 3, with coefficients statistically significant (*p* < .05) [Color figure can be viewed at http://wileyonlinelibrary.com]

Figure 11 shows the aging trajectories of the *Myelin‐Nets* global network properties (NPs) for a period of 27 years from 48 years old to 75 years old. The best polynomial fit of the age trajectories was obtained with order *n* = 3, (based on AIC) for all NPs. In each case the polynomial coefficients were statistically significant (*p* < .05). The clustering index and local efficiency showed (Figure 11 panel a and b, respectively) an inverted U‐shape aging trajectory. The minimum value for both was found at 48 years old, and the maximum around 60 years and 60–65 years for the clustering index and local efficiency, respectively.

On the other hand, the characteristic path length (Figure 11, panel c) showed a minimum at 48 years old and a maximum around 55 years. The age trajectory of the global efficiency (Figure 11, panel d) showed a U‐shape with a minimum peak between 55 and 60 years old and a maximum value at 48 years old. Moreover, the global connectivity (Figure 11, panel e) was minimum at 48 years old with a maximum peak between 55 and 60 years old, whereas the strength of correlation between homologous regions (Figure 11, panel F) showed a monotonic increasing behavior starting at 48 years old.

### 3.9 Aging trajectory of the precuneus and posterior cingulate gyrus: structures with highest NBC in young and old age groups

**Figure 12:**
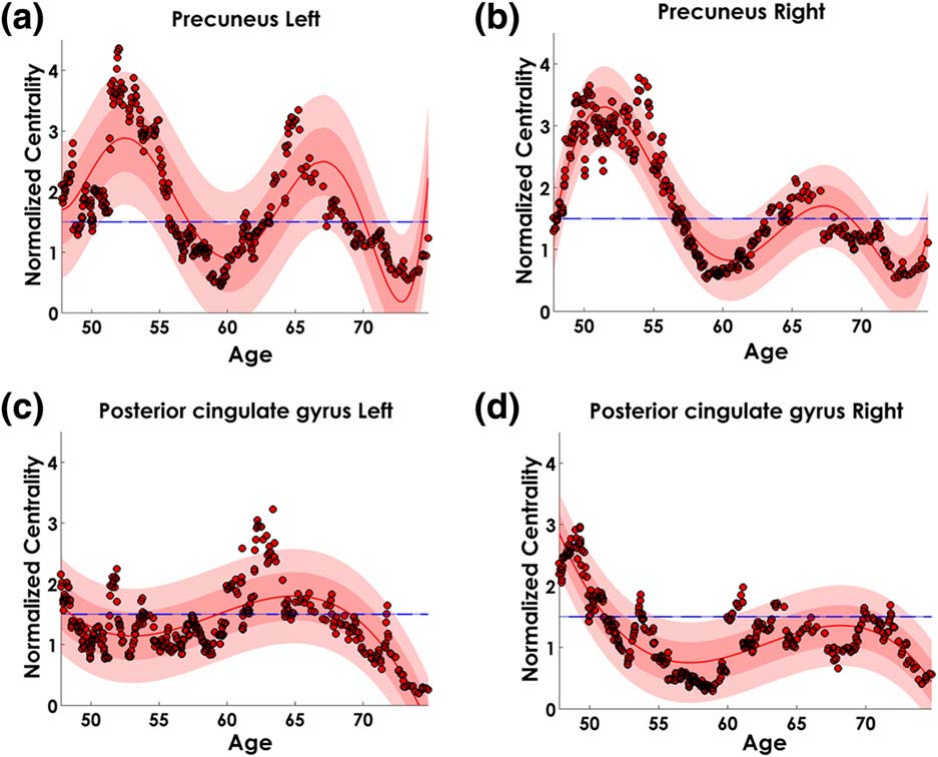
Age trajectory of the normalized betweenness centrality (NBC) in the Precuneus (PCu) (panels a and b) and Posterior cingulate gyri (PCgG) (panels c and d) structures. The continuous line in red represents the polynomial fitted function. In all cases the polynomial coefficients were statistically significant (*p* < .05). The light red shaded area shows the confidence interval of the polynomial fit, and the dark shaded area the standard deviation of the error in predicting a future observation. Dots in red and black represent the NBC values for each “age”—taken as the median age of the participants in the particular window. Line in blue at NBC = 1.5 shows the NBC threshold for which a region is considered as hub [Color figure can be viewed at http://wileyonlinelibrary.com]

Here we focused our attention (Figure 12) on the NBC age trajectory of the precuneus left (PCu.L) and left posterior cingulate gyrus (PCgG.L), the regions with the highest NBC in the Young‐Age and Old‐Age groups, respectively (see Figure 7, panel b). We also show the age trajectory of the contralateral homologous structures.

The Precuneus shows a nonmonotonic and complex temporal dynamic (best polynomial order n = 6, based on AIC), with two principal maxima between 50–55 years and 65 years old (Figure 12, panel a and b). The PCu.L and PCu.R NBC minima were approximately at 60 and 73 years with a NBC < 1.5. The NBC in both precunei is reduced at the second maxima as compared with the former peak.

On the other hand, PCgG.R and PCgG.L (Figure 12, panel c and d) showed less complex trajectory (best polynomial order *n* = 3, based on AIC) with maxima around 50 and 65 years for PCgG.R right and PCgG.L, respectively.

### 3.10 Influence of the gray matter parcellation over aging effects of Myelin‐Nets global network properties

**Figure 13:**
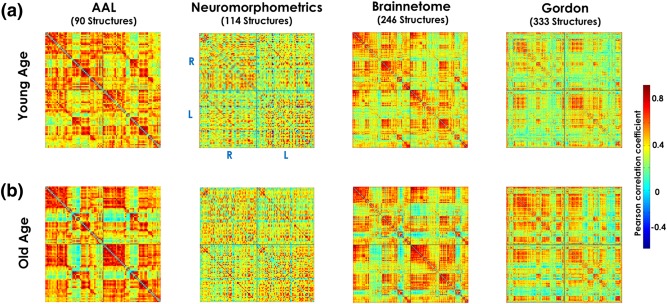
Correlation matrices associated to *Myelin‐Nets* for the Young Age (panel a) and Old Age (panel b) groups for the different gray matter parcellations (AAL, Neuromorphometrics, Brainnetome and Gordon atlases). In panel a (similar to all plotted correlation matrices), the “R–R” and “L–L” quadrants represent the intrahemispheric myelin correlations in the right and left hemispheres, respectively. The “R–L” and “L–R” quadrants depict the interhemispheric interactions. The diagonal of the “L–R” quadrant shows the correlations in myelination between homologous structures across hemispheres. The color bar on the right represents the scale of the Pearson correlation coefficients using a “jet” color map [Color figure can be viewed at http://wileyonlinelibrary.com]

Figure 13 shows the correlation matrices associated to *Myelin‐Nets* for the Young‐Age (panel A) and Old‐Age (panel B) groups and the different gray matter parcellations (AAL, Neuromorphometrics, Brainnetome, and Gordon atlases).

Table 3 shows the summary of the network property (AUC) statistics and targeted attack study, comparing both age groups, for the different atlas parcellations. More details about the statistics results can be found in Supporting Information, Tables S5–S7. The AAL‐based parcellation did not yield group differences in any of the topological network properties and depicted the lowest statistical sensitivity of all the parcellations. Interestingly, the AAL atlas was also reported to yield the lowest homogeneity in resting state activity in Gordon's study (Gordon et al., 2016). In contrast, the Neuromorphometrics atlas provided the highest sensitivity to differences between age groups in all NPs except for the global connectivity attribute. It is important to point out that clustering index, characteristic path length, and local and global efficiency were the most reproducible measures across brain parcellations. Another relevant and consistent result is that global connectivity showed no significant differences between age groups independently of the brain parcellation.

**Table 3:** Summary of Network properties (AUC) statistics and Targeted attack study comparing both age groups for different atlas parcellations [Color table can be viewed at http://wileyonlinelibrary.com]

Network property	AAL atlas (Nstruct = 90)	Neuromorphometric atlas (Nstruct = 114)	Brainnetome atlas (Nstruct = 244)	Gordon atlas (Nstruct = 333)
Clustering index	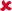	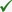	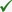	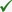
Characteristic path length	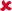	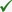	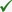	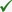
Local efficiency	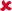	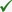	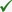	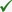
Global efficiency	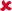	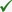	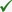	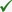
Global connectivity	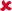	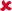	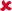	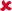
Targeted attack	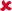	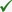	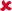	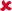
Homologous regions connectivity	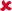	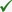	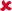	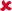

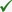
: Significant differences between Young and Old Age groups (*p* < .05, CI 95% does not contain zero); 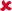: nonsignificant differences between Young and Old Age groups (*p* > .05, CI 95% contains zero).

## 4 DISCUSSION

In this study, we provide empirical evidence that myelination processes in gray matter are spatially correlated, irrespective of the distance between brain regions. When pooling together all possible covariations between pairs of structures, a nonrandom network of concurrent changes emerged: the network of myelination covariance (*Myelin‐Net*). The *Myelin‐Net* showed well‐structured topological patterns strongly modulated by age. Our findings can be summarized as follows: (a) Aging modulates the correlation strength between homologous regions but not the global myelin correlation strength. (b) The topological attributes of the *Myelin‐Nets* depict a strong aging modulation. (c) Aging shapes the distribution of the central regions (hubs) of the *Myelin‐Nets*. (d) The aging process increases the resilience of the *Myelin‐Nets* to damage to the network's hubs. These findings are discussed in the following subsections.

### 4.1 Correlated myelination processes in the brain: Possible mechanisms

In this article, we found that myelination fluctuates concurrently across the brain gray matter with well‐defined patterns. Several mechanisms have been proposed to explain the concurrent fluctuations between the spatially distinct regions of the networks of anatomical covariance: (a) common normal/pathological vulnerabilities; (b) neurodevelopment; (c) genetic factors (Alexander‐Bloch et al., 2013); and (d) the presence of axonal connections between brain structures (Gong, He, Chen, & Evans, 2012). We hypothesize that these putative mechanisms may explain the synchronicity of myelination processes between gray matter regions.

One could expect that if two brain structures are anatomically connected by nervous fibers, and if the density of myelin along the fiber trajectories is relatively constant and distributed across axonal branches inside each region, these distant structures might show correlated myelin densities. If a proportion of nervous fibers were to degenerate, myelin density in the gray matter structures connected by these fibers might concurrently decrease. Alternatively, intracortical myelin changes may act to optimize synchronicity and timing between structures and their reciprocal connections (Haroutunian et al., 2014). These homeostatic mechanisms may be responsible for correlated myelination processes, acting to preserve an optimal conduction delay (timing) and thus synchrony of neural information processing between connected structures (Kimura and Itami, 2009; Salami, Itami, Tsumoto, & Kimura, 2003). Hunt et al. (2016) and Huntenburg et al. (2017) have recently provided empirical evidence in support of this idea. The former showed that the occurrence of concurrent myelination changes between brain anatomical structures has a high predictive value of electrophysiological functional connectivity. Moreover, Huntenburg et al. demonstrated that functional connectivity (using fMRI) is higher between brain areas with similar intracortical myelin levels. Therefore, if two regions are axonally wired and so functionally coupled, myelination correlations are necessarily induced: anatomical connectivity, myeloarchitecture, and functional networks are intimately linked.

Other factors that could be playing an important role are the shared vulnerability of gray matter regions and neural compensation by normal and abnormal processes. It is well known that myelination is highly dynamic and can be modulated by experimental manipulations and environmental factors (Chang, Redmond, & Chan, 2016; Fields, 2005; Yeung et al., 2014). The Scaffolding Theory of Aging and Cognition (STAC) model (Reuter‐Lorenz and Park, 2014) is based on this observation and attempts to explain how the brain reacts to different challenges. This theory represents the brain as a dynamically adaptive structure that changes in both positive and negative ways with age. During neurodevelopment and normal aging, myelination processes are expected to correlate among groups of structures as a consequence of the establishment and consolidation of neural circuits in the former case, and to compensate aging‐related neural degradations (i.e., cortical thinning, myelin deterioration, etc.) in the latter. It is known that the degradation of myelin (presence of balloons, etc.) across the lifespan triggers continuous remyelination as repair mechanism (Peters, 2002). This phenomenon can be thought of as one that would increase correlations in myelination between structures if they are involved in the same pathological events. Also the need to recruit additional neural resource to sustain cognitive performance in middle‐age and old‐age brains increases the chance of new myelination correlations to emerge.

On the other hand, there is no doubt that genetics is a key player influencing concurrent changes between regions in morphometric, functional, and tissue properties (Alexander‐Bloch et al., 2013; Brown et al., 2011; Goryawala et al., 2015; Shu et al., 2015; Yao et al., 2015). Twin studies have demonstrated that specific anatomical co‐variations rest on shared genetic influences (Alexander‐Bloch et al., 2013; Schmitt et al., 2009, 2010, 2008). Myelination processes could be modulated by shared genetic influences due to a single gene, as a putative mechanism of synchronization of these processes among brain regions during neurodevelopment and aging. This mechanism has previously been proposed studying other brain anatomy variables (Meyer‐Lindenberg et al., 2007; Pezawas et al., 2005). Along the same line of thought, the interaction of different genes involved in brain myelination (related to myelin structure, composition, development, or maintenance; Jungerius et al., 2008) may contribute to correlations in myelination between regions.

Further experimental work (animals and humans, in vivo and ex vivo) and theoretical modelling need to be conducted to support or disprove the impact of these putative factors and mechanisms on correlations in myelination between gray matter regions.

### 4.2 Concurrent myelin changes in the brain: Aging effects

The study of myelination processes is crucial to understand brain anatomy and function. Here we extended the standard analysis of exploring myelin distributions across the gray matter (first‐order analysis) to a second order approach, where the amount of myelin in each anatomical region is no longer relevant (the mean regional myelination values were detrended out before analysis). It is this approach that allows the estimation of the interactions between structures by the Pearson correlation coefficients. It has been proven that such type of analysis reveals hidden organizational properties of brain anatomy that are difficult to detect through classical univariate approaches (Bassett et al., 2012; Li, Bin, Hong, & Gao, 2010; Melie‐García et al., 2013; Sánchez‐Catasús et al., 2017; Yu et al., 2013; Zalesky, Fornito, Egan, Pantelis, & Bullmore, 2012).

Interestingly, we found that concurrent gray matter myelination processes are largely shaped by age. The global correlation strength—an aggregate measure of the correlation values between all possible pairwise structures—showed no significant change between age groups, suggesting that at the global level, mechanisms underlying demyelination and neurodevelopment in Old‐age and Young adults, respectively, are similarly coordinated across subjects. However, the local myelination correlations in brain lobes were different between age groups. This suggests that multiple myelination mechanisms exist and that the balance of their expression is diverse. For instance, temporal, parietal, and occipital lobes behaved similarly to the global pattern, showing no modulation with age. The other lobes showed higher correlation strength for the Old‐Age group, suggesting that the myelination mechanisms characteristic of old age are more homogeneous across the aged brain. On the other hand, the aging trajectory of the global correlation strength from middle age (>48 years) onwards showed a minimum at 48 years (Figure 11, panel E). This could be explained by the variability of the peak myelination age across structures and subjects around 48 years old (Supporting Information, Figure S2) that affects the synchronization of the myelination inter‐individual differences and consequently induces lower correlation strengths between anatomical structures.

It is important to point out the observed strong age modulation in myelination covariance between homologous regions. The covariance of homologous structures was found to increase significantly with age. This suggests that interindividual differences in myelination due to neurodevelopmental factors are less coordinated than the putative compensatory, demyelination/remyelination and shared vulnerability processes that become prominent in later life. Myelination changes in old age are noticeably more synchronized and spatially extended than in young age, in line with the scaffolding hypothesis of the STAC theory of aging (Reuter‐Lorenz and Park, 2014). More evidence was found in the monotonically increasing aging trajectory of the myelination covariance between homologous regions (Figure 11, panel F) from 48 years old onwards. It is interesting to consider these results in the light of the known increase in symmetry in brain function and anatomy with age expressed by the recruitment of homologous areas, which may operate to compensate for cognitive decline (Cabeza et al., 1997; Cabeza, 2002; Grady, McIntosh, Rajah, Beig, & Craik, 1999; Park and Reuter‐Lorenz, 2009).

We also observed that the highest correlations were precisely between homologous regions for both age groups. This finding has been systematically reported in the literature using different functional and anatomical variables (Eyler et al., 2011; Mechelli et al., 2005; Melie‐García et al., 2013; Sanabria‐Diaz, Martínez‐Montes, & Melie‐Garcia, 2013; Schmitt et al., 2009). We hypothesize that genetic factors play an important role in fine‐tuning the correlation of the myelination processes between homologous structures.

### 4.3 Most connected structures in Myelin‐Net, Myelin‐Nets hubs, and aging effects

To better understand the topological organization of spatial myelination changes, we identified the structures where myelination processes are the most correlated with the myelination variations across the rest of the brain. Some structures were common to both age groups (e.g., bilateral supplementary motor cortex (SMC.L, SMC.R) and left superior parietal lobe (SPL.L)). The presence of SMC as a network hub has been described previously at different ages. In the case of the elderly connectome (76–94 years), this structure is preserved as a hub (Hwang, Hallquist, & Luna, 2013; Perry et al., 2015).

We evidenced a redistribution of hubs with aging (Figure 7). There were 12 hub regions common to both groups. This result indirectly reveals how the flux of information is reorganized by aging in the *Myelin‐Nets*. Along the same line, we found a profound age modulation of the NBC (which objectively quantifies the central role of a region in our *Myelin‐Nets*) in Precuneus Left (PCu.L) and Left Posterior cingulate gyrus (PCgG.L) structures (amongst the coincident hub structures, PCu.L and PCgG.L showed the highest NBC in Young and Old age groups respectively). The NBC of PCu.L/PCgG.L was lower/higher in the Old‐Age group. Both regions are part of the Default Mode Network and impose high metabolic demand on the brain (Buckner, Andrews‐Hanna, & Schacter, 2008). It has been consistently shown that normal aging induces a disruption of the connectivity and hubs within the DMN, more specifically along the anteriorposterior axis of the network (Andrews‐Hanna et al., 2007; Biswal et al., 2010; Grady et al., 2010; Jones et al., 2011; Meunier, Achard, Morcom, & Bullmore, 2009; Preisig et al., 2009; Wu et al., 2011a)

### 4.4 Organizational properties and aging effects of myelin nets

The topological properties of the *Myelin‐Nets* were also modulated by aging. The “characteristic path length” in Old‐Age was statistically higher than the Young‐Age group. This alteration has been reported consistently in previous studies using other neuroimaging modalities (Gong et al., 2009b; Petti et al., 2016; Zhao et al., 2015; Zhu et al., 2012). The nature of this effect is unknown but could be related to compensatory effects and common pathological processes or shared vulnerability. These processes could influence the redistribution of principal myelin covariations (the highest correlation values) to specific regions, which ultimately may induce longer characteristic path lengths. Additionally, we provide empirical evidences that after 48 years old this network attribute present dynamic changes until 75 years old.

The “clustering index” was increased in the Old‐Age group. This is a measure of the similarity of myelin covariations among brain structure neighborhoods (not reduced to a physical neighborhood concept). According to the graph theory, this increase could be generated by the establishment of new densely connected local clusters which may generate an uncontrolled “flow of information” through the entire network. This measure is related to the local efficiency of the “information flow” of the networks and its abnormal performance could be attributed to shared vulnerability and compensatory mechanisms. The increased intralobe myelin covariation in 4 out of 7 lobes might explain the larger clustering index in old age.

The increasing “local efficiency” was accompanied by a “global efficiency” decline in Old‐Age. In terms of the graph theory, these changes affect network performance pointing to a higher “wiring cost” for parallel “information transfer” between anatomical regions. The weakening of the global efficiency is explained by the larger characteristic path length present in old age.

The aging trajectory in both “clustering index” and “local efficiency” showed a minimum at 48 years old that is related to global myelin correlation strength changes and the cause producing that this attribute has a minimum value at the same age. The maxima of these two network properties and the maximum/minimum in characteristic‐path‐length/global‐efficiency are synchronized in some extend revealing an unbalance between segregation and integration processes in *Myelin‐Nets*.

In our “Targeted Attack” analysis, we attempted to further investigate the age‐associated changes in the resilience of the *Myelin‐Nets*. Networks were “attacked” at key nodes of the highest betweenness centrality (network hubs). We found that the Old‐Age group showed higher resilience to these simulated attacks. A possible explanation for this finding may rest on the observed increase in symmetry and the presence of aberrant local circuits leading to increased “local efficiency” with age. Also the redistribution and the higher number of hubs in the Old‐Age group favored a higher resilience to target damage. Attacks to hub regions in the Young‐Age group produced devastating consequences in the integrity and stability of the network, where regional specialization is well defined.

The comparison of the networks of myelin reported in this study and Networks of anatomical covariance may bring new light on the interplay between tissue myelination and morphological changes in the brain. Our results indicate that *Myelin‐Nets* and Networks of anatomical covariance have common topological features. For both brain networks, correlations between homologous regions are the highest and are modulated by age. Also, the global network attributes—characteristic path length, clustering index, and local efficiency—share the same age modulation across networks (Chen et al., 2011; Wu et al., 2012; Zhu et al., 2012). Additionally, *Myelin‐Nets* and Networks of anatomical covariance share common hub regions such as precuneus, middle and superior frontal gyrus, prefrontal cortex, posterior cingulate cortex, inferior temporal gyrus, and medial temporal gyrus (He et al., 2007; Wu et al., 2012). These similarities suggest possible common principles driving the topological organization of the synchronized myelination and morphological changes across the gray matter. However, a number of differences are also apparent which might be due in part to differences in the data and analysis methods used, which motivates further investigation to be conducted using the same experimental conditions (same subjects sample, brain parcellation, weighted/binary graphs, etc.) to ease the comparability of the results.

Finally, a comparison between *Myelin‐Nets* and white matter structural networks (*WM‐Nets*) would shed light on the common principles driving the correlated myelination processes and anatomical (axonal) connectivity. A separate study, preferably based on *Myelin*‐ and *WM‐Nets* extracted from the same cohort, is necessary to highlight the interplay between these processes. However, some similarities and differences can be highlighted in light of the main anatomical connectivity findings reported in the literature. First, in both myelin and anatomical networks, the precuneus and posterior cingulate gyrus are observed as centrally connected regions, independently of age (Gong et al., 2009a, 2009b; Iturria‐Medina, Sotero, Canales‐Rodríguez, Alemán‐Gómez, & Melie‐García, 2008). This observation is consistent with the study of (Hagmann et al., 2008), who identified a structural core within posterior medial and parietal cortex in the cortical anatomical network. A region equivalent to the precuneus was also observed as a hub in the macaque cortical network (Sporns, Honey, & Kötter, 2007). Also, putamen and superior parietal structures were identified as most vulnerable areas in *WM‐Nets* (Iturria‐Medina et al., 2008) as we found in *Myelin‐Nets*.

The topological efficiency of the WM‐Nets exhibits an inverted U‐shaped trajectory across the lifespan, peaking around the third decade of age (Zhao et al., 2015). Similarly, we found that the local efficiency of the *Myelin‐Nets* followed an inverted U‐shaped between the ages of 48 and 75 years old, but with a peak between 60 and 65 years old. In contrast, the global efficiency of the *Myelin‐Nets* was found to follow a U‐shape with a minimum between 55 and 60 years. While the clustering index and characteristic path length of *WM‐Nets* were found to follow an inverted U‐shaped trajectory (Zhao et al., 2015), these topological attributes were found to exhibit different aging trajectories in the *Myelin‐Nets* in the age range 48–75 years. Interestingly, while the WM‐Nets become less connected with age (Gong et al., 2009b), the connectivity in *Myelin‐Nets* shows a tendency to increase with age from 48 years old onward.

### 4.5 Further considerations and future work

Some issues should be addressed in future works. We propose the use of parcellations closer to the myelo‐architectonic organization of the cortex to study *Myelin‐Nets* topological properties. The Neuromorphometrics, AAL, Brainnetome, and Gordon atlases employed in this study do not take into account this key organization feature of the cerebral cortex. Partial correlation, instead of the Pearson correlation, should be favored to compute the interaction between anatomical structures accounting for the effect of other structures and possible global variables. An increase in the number of subjects per age group in an independent dataset would provide evidence for the reproducibility of our results.

A natural extension of the presented work is the parallel characterization of morphological and myelin covariance networks in gray matter. This combined study, combining micro and mesoscopic measures of the brain, would shed a new light into the biophysical mechanisms underlying the emergence of such networks. It should be highlighted that myelin is the primary contributor to MR image contrast (Geyer, Weiss, Reimann, Lohmann, & Turner, 2011), with a clear impact on local gray matter volume estimates (Helms et al., 2009; Lorio et al., 2016). The parallel study of morphological and myelination change would therefore allow disambiguating true morphological brain changes and spurious changes arising from changes in myelination.

An important line of future research is the exploration of potential deviations in the correlation of myelination processes in pathologies like Alzheimer, Schizophrenia, Multiple Sclerosis, and Epilepsy. Furthermore, the combined study of anatomical, myelin, functional brain networks, by combining structural DWI, magnetization transfer‐MRI, and functional MRI techniques in a same set of subjects would be tremendously useful for discovering similarities and differences to brain network properties obtained from different physiological variables. This would help to understand how changes in the topological organization of myelination processes are related to brain function. The study of the association between gray and white matter myelination processes over the lifespan will help to improve our knowledge about the basic principles of the brain topological organization. Finally, we find two potential limitations of our methodology: (a) some of the identified age‐related differences in *Myelin‐Net* topological organization may be because of possible registration errors in the Neuromorphometrics atlasing labeling methodology. However, these sources of bias were minimized by using a nonlinear advanced registration algorithm (geodesic shooting registration; Ashburner and Friston, 2011) and a probabilistic labeling approach. The influence of our results by the utilization of surface‐based and volume‐based registration algorithms should be evaluated in future studies. (b) Although myelination largely dominates the MT contrast other processes such as inflammation (Bélanger, Allaman, & Magistretti, 2011; Gloor, Scheffler, & Bieri, 2008), metabolism (Giulietti et al., 2012), and pH changes (Gillies, Raghunand, Garcia‐Martin, & Gatenby, 2004; Gloor et al., 2008; Kucharczyk, Macdonald, Stanisz, & Henkelman, 1994) appear to contribute to this signal (Harrison et al., 2015). The neuroinflammation, for instance, is present during aging (Chung et al., 2009; Franceschi et al., 2007); therefore, our results may be influenced by the heterogeneity of this process in our age groups.

## 5 CONCLUSIONS

In this article, we provide empirical evidence that myelination processes are spatially correlated across cortical gray matter. The patterns of myelination covariance show specific topological organization revealed using a graph theoretical approach. A number of specific correlated myelination phenomena were strongly modulated by age. In particular, we brought evidence that aging increases synchronicity between homologous regions. Our results are an important step toward elucidating the organizational principles behind the dynamics of the human brain anatomy across the lifespan. In particular, myelination processes are at the cross‐way of several psychiatric and neurodegenerative brain diseases and are therefore crucial for their understanding.

## CONFLICT OF INTEREST

The authors have declared that no conflict of interests exists.

## Supporting information

Additional Supporting Information may be found online in the supporting information tab for this article.

Supporting Information
