# Supplementary material for: Networks of myelin covariance
Source: Hum Brain Mapp. 2017 Dec 21;39(4):1532–54. doi: 10.1002/hbm.23929 (PMC5873432; doi:10.1002/hbm.23929)
Supplement: Supplementary file 1 — Supporting Information [file HBM-39-1532-s001.docx]

**SUPPLEMENTARY MATERIAL**

**Networks of Myelin covariance**

Lester Melie-Garcia^1†^, David Slater^1^, Anne Ruef^1^, Gretel Sanabria-Diaz^1^, Martin Preisig

Ferath Kherif^1^, Bogdan Draganski^1^, Antoine Lutti^1^

*^1^LREN, Department of Clinical Neurosciences, Lausanne University Hospital (CHUV). Switzerland*

*^2^Department of Psychiatry, Lausanne University Hospital (CHUV), Switzerland*

*^3^Max-Planck-Institute for Human Cognitive and Brain Sciences, Leipzig, Germany*


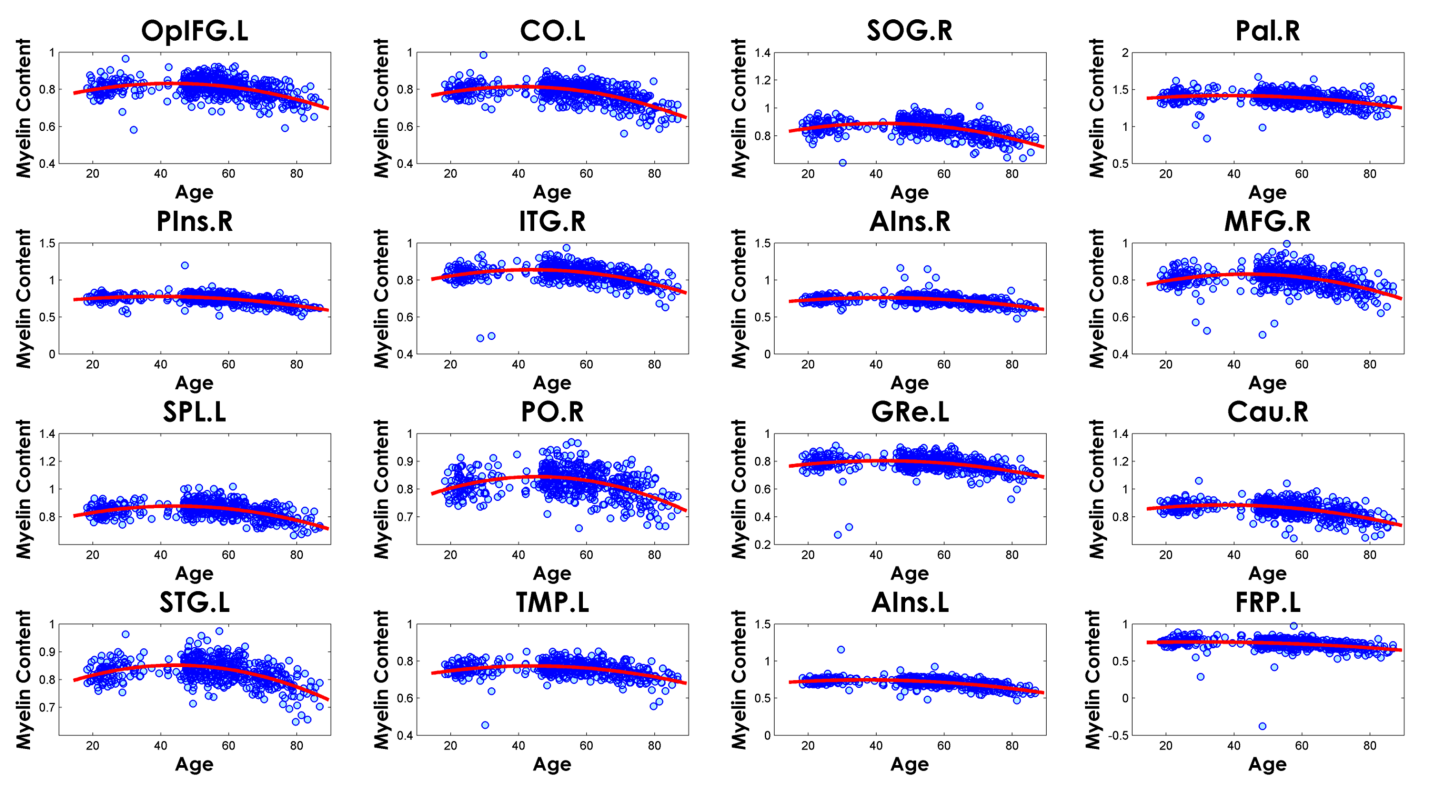


**Figure S1.** Plot of the MT values (as a surrogate of gray matter myelin content) for 562 subjects versus age (18–87 years) in a subset of 16 anatomical structures. It is observed an inverted-U shape trajectory of myelin changes with age in all regions. The peak of myelination is achieved between 40 and 50 years. Aging has a heterogeneous effect over myelination across gray matter structures. The full name of structures can be found in Table S1.

**
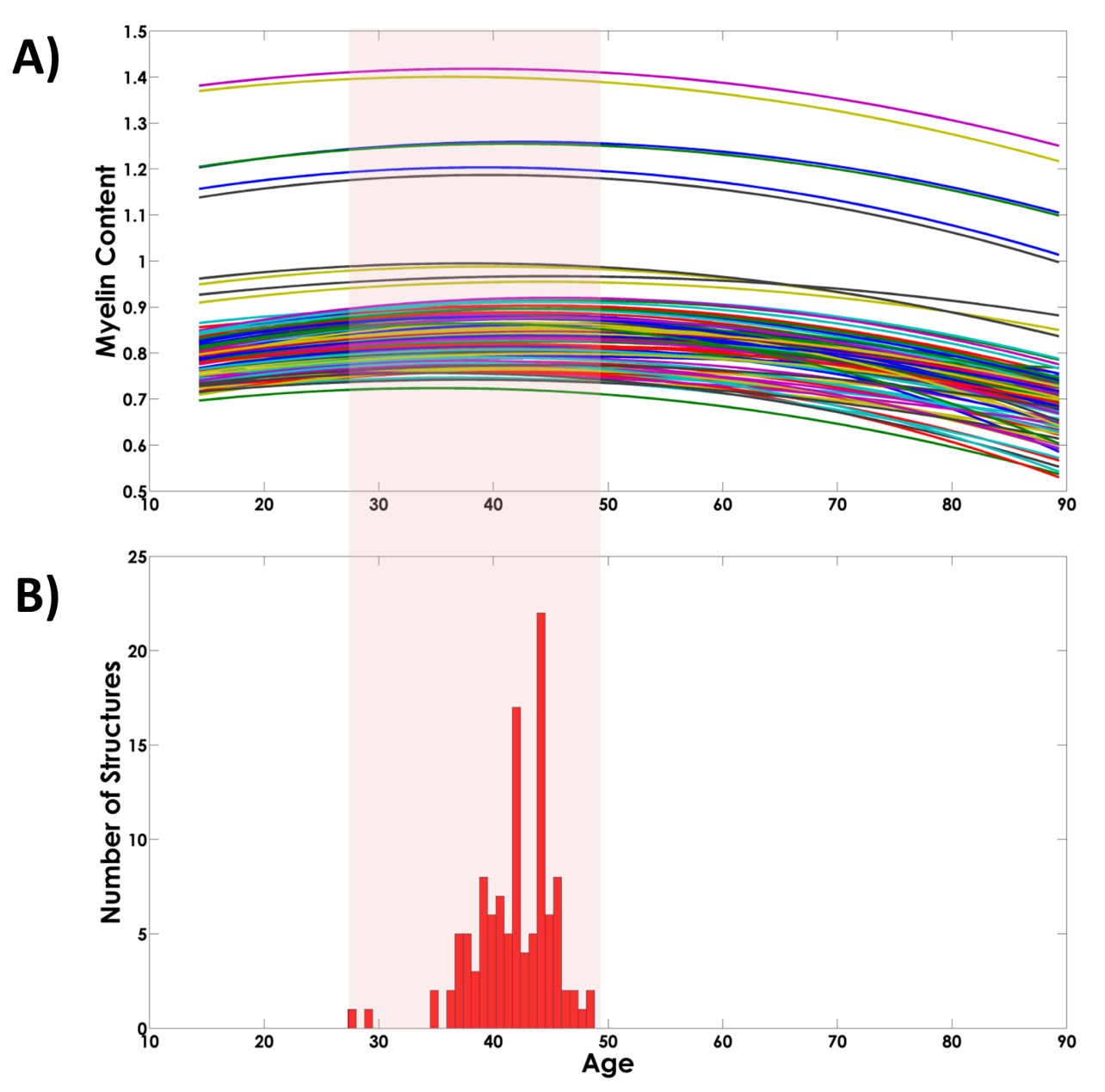
**

**Figure S2.** Panel A) Plot of the fitted second order polynomial function of MT values (as a surrogate of gray matter myelin content) for all anatomical structures. Panel B) Histogram showing the myelination peak age of the 114 anatomical structures. This counts the number of structures with a specific myelination peak age. The peak of myelination is achieved between 40 and 50 years for most of brain anatomical regions. The maximum number of structures (more than 20) have its myelination peak close to 45 years old.

**
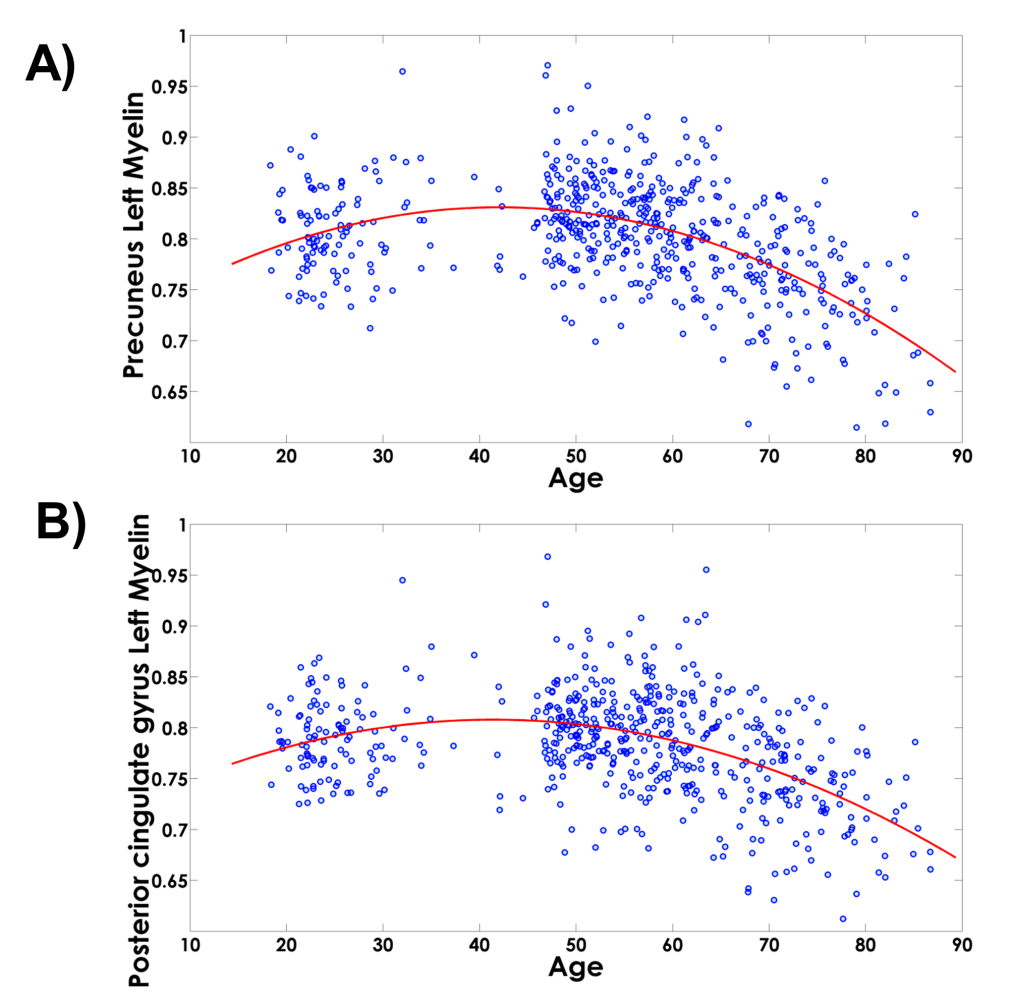
**

**Figure S3.** Plot of the fitted second order polynomial function of MT values (as a surrogate of gray matter myelin content) for Precuneus Left (PCu.L) (Panel A) and Left Posterior Cingulate gyrus (Panel B).

**
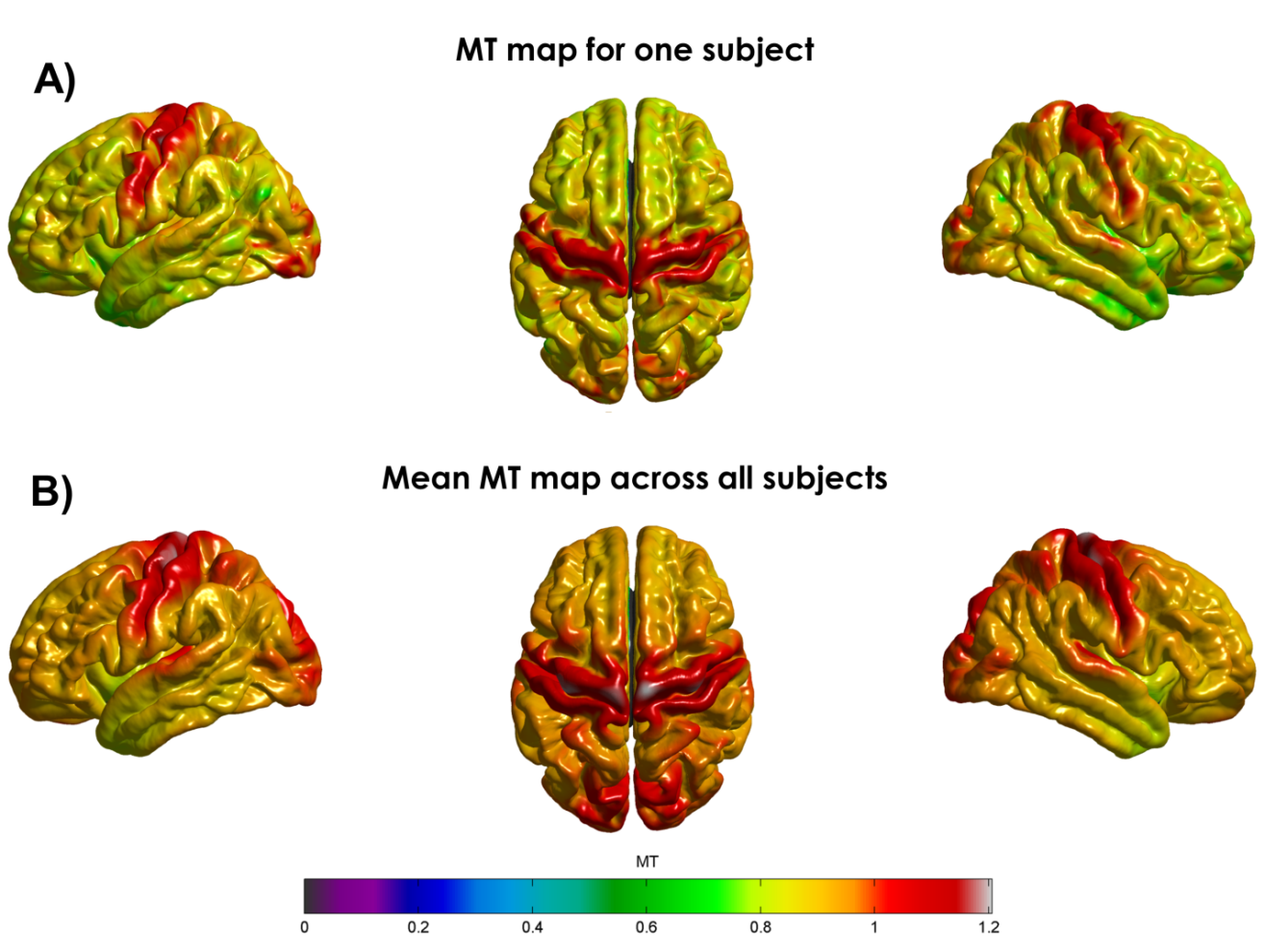
**

**Figure S4.** A) Plot of the MT map for a specific subject of our cohort. B) Mean MT across all subjects of the sample. It is observed, as has been reported in previous studies, the primary brain regions are the most myelinated.

**Table S1**. List of gray matter structures defined in the Neuromorphometrics atlas used in this paper.

| Structure Name | Abbreviated Name Left | Abbreviated Name Right |
| --- | --- | --- |
| Accumbens Area | Accum.R | Accum.L |
| Amygdala | Amyg.R | Amyg.L |
| Caudate | Cau.R | Cau.L |
| Hippocampus | Hip.R | Hip.L.L |
| Pallidum | Pal.R | Pal.L |
| Putamen | Put.R | Put.L |
| Thalamus Proper | Thal.R | Thal.L |
| Ventral DC | VentDC.R | VentDC.L |
| Anterior cingulate gyrus | ACgG.R | ACgG.L |
| Anterior insula | AIns.R | AIns.L |
| Anterior orbital gyrus | AOrG.R | AOrG.L |
| Angular gyrus | AnG.R | AnG.L |
| Calcarine cortex | Calc.R | Calc.L |
| Central operculum | CO.R | CO.L |
| Cuneus | Cun.R | Cun.L |
| Entorhinal area | Ent.R | Ent.L |
| Frontal operculum | FO.R | FO.L |
| Frontal pole | FRP.R | FRP.L |
| Fusiform gyrus | FuG.R | FuG.L |
| Gyrus rectus | GRe.R | GRe.L |
| Inferior occipital gyrus | IOG.R | IOG.L |
| Inferior temporal gyrus | ITG.R | ITG.L |
| Lingual gyrus | LiG.R | LiG.L |
| Lateral orbital gyrus | LOrG.R | LOrG.L |
| Middle cingulate gyrus | MCgG.R | MCgG.L |
| Medial frontal cortex | MFC.R | MFC.L |
| Middle frontal gyrus | MFG.R | MFG.L |
| Middle occipital gyrus | MOG.R | MOG.L |
| Medial orbital gyrus | MOrG.R | MOrG.L |
| Postcentral gyrus medial segment | MPoG.R | MPoG.L |
| Precentral gyrus medial segment | MPrG.R | MPrG.L |
| Superior frontal gyrus medial segment | MSFG.R | MSFG.L |
| Middle temporal gyrus | MTG.R | MTG.L |
| Occipital pole | OCP.R | OCP.L |
| Occipital fusiform gyrus | OFuG.R | OFuG.L |
| Opercular part of the inferior frontal gyrus | OpIFG.R | OpIFG.L |
| Orbital part of the inferior frontal gyrus | OrIFG.R | OrIFG.L |
| Posterior cingulate gyrus | PCgG.R | PCgG.L |
| Precuneus | PCu.R | PCu.L |
| Parahippocampal gyrus | PHG.R | PHG.L |
| Posterior insula | PIns.R | PIns.L |
| Parietal operculum | PO.R | PO.L |
| Postcentral gyrus | PoG.R | PoG.L |
| Posterior orbital gyrus | POrG.R | POrG.L |
| Planum polare | PP.R | PP.L |
| Precentral gyrus | PrG.R | PrG.L |
| Planum temporale | PT.R | PT.L |
| Subcallosal area | SCA.R | SCA.L |
| Superior frontal gyrus | SFG.R | SFG.L |
| Supplementary motor cortex | SMC.R | SMC.L |
| Supramarginal gyrus | SMG.R | SMG.L |
| Superior occipital gyrus | SOG.R | SOG.L |
| Superior parietal lobule | SPL.R | SPL.L |
| Superior temporal gyrus | STG.R | STG.L |
| Temporal pole | TMP.R | TMP.L |
| Triangular part of the inferior frontal gyrus | TrIFG.R | TrIFG.L |
| Transverse temporal gyrus | TTG.R | TTG.L |

**Table S2.** Intra Lobe correlation statistics comparing the two age groups.

| Lobe | Group 1  (Young Age) | Group 2  (Old Age) | Confidence Interval (95%) |
| --- | --- | --- | --- |
| Frontal | 0.59 (0.046) | 0.69 (0.031) | **(-0.21,-0.018)** |
| Insula | 0.26 (0.134) | 0.63(0.077) | **(-0.72,-0.36)** |
| Limbic | 0.42 (0.086) | 0.68 (0.038) | **(-0.43,-0.10)** |
| Subcortical Nuclei | 0.60 (0.156) | 0.87 (0.016) | **(-0.55,-0.04)** |
| Occipital | 0.71 (0.055) | 0.82 (0.031) | (-0.25, 0.007) |
| Parietal | 0.67 (0.075) | 0.76 (0.043) | (-0.28,0.061) |
| Temporal | 0.52 (0.041) | 0.53 (0.065) | (-0.157,0.14) |

Connectivity in each Lobe is represented by the mean and standard deviation: mean (standard deviation).

In bold the significance differences: those confidence intervals that do not contain zero.

**Table S3.** Statistical results of comparing the Normalized Betweenness Centrality (NBC) in the two most important hubs common to both groups. Precuneus left (PCu.L) is the hub with the highest NBC for Young Aging group, while Left Posterior cingulate gyrus (PCgG.L) for Old Aging group.

| Structure | Group 1  (Young Age) | Group 2  (Old Age) | Confidence Interval (95%) |
| --- | --- | --- | --- |
| Precuneus Left (PCu.L) | 3.32 (0.93) | 1.65 (0.78) | **(0.25, 5.49)** |
| Left Posterior cingulate gyrus (PCgG.L) | 1.51 (0.61) | 2.58 (0.93) | **(-5.21, -0.37)** |

NBC is represented by the mean and standard deviation: mean (Standard deviation). In bold the significance differences: those confidence intervals that do not contain zero.

**Table S4.** Network properties (AUC) statistics and Targeted attack study comparing both age groups using Neuromorphometrics atlas parcellation (114 anatomical structures).

| Network Property | Group 1  (Young Age) | Group 2  (Old Age) | Confidence Interval (95%) |
| --- | --- | --- | --- |
| Clustering Index | 10.41 (0.284) | 11.13 (0.30) | **(-2.35,-0.95)** |
| Characteristic Path Length | 30.09 (0.32) | 30.83 (0.44) | **(-4.55,-0.146)** |
| Local Efficiency | 13.53(0.13) | 13.97 (0.16) | **(-1.49,-0.81)** |
| Global Efficiency | 10.99 (0.069) | 10.88 (0.069) | **(0.12,0.54)** |
| Global Connectivity | 0.36 (0.033) | 0.37 (0.034) | (-0.11,0.079) |
| Targeted Attack | 17.71 (0.64) | 18.72 (0.58) | **(-3.14,-0.17)** |
| Homologous regions connectivity | 0.58 (0.15) | 0.71 (0.11) | **(-0.43,-0.11)** |

Network properties in each group are represented by the mean and standard deviations: mean (Standard deviation).

In bold the significance differences, those confidence intervals that do not contain zero.

**Table S5.** Network properties (AUC) statistics and Targeted attack study comparing both age groups using AAL atlas parcellation (90 anatomical structures).

| Network Property | Group 1  (Young Age) | Group 2  (Old Age) | Confidence Interval (95%) |
| --- | --- | --- | --- |
| Clustering Index | 10.87(0.23) | 11.43 (0.32) | (-1.55,0.026) |
| Characteristic Path Length | 30.96(0.39) | 32.39(0.97) | (-4.34,-0.05) |
| Local Efficiency | 13.82(0.11) | 13.95(0.20) | (-0.67,0.19) |
| Global Efficiency | 10.88(0.070) | 10.60(0.14) | (0.002,0.63) |
| Global Connectivity | 0.487(0.041) | 0.49(0.044) | (-0.11,0.12) |
| Targeted Attack | 14.88(0.40) | 14.55(0.59) | (-0.82,1.94) |
| Homologous regions connectivity | 0.75 (0.021) | 0.75(0.028) | (-0.072,0.073) |

Network properties in each group are represented by the mean and standard deviations: mean (Standard deviation).

In bold the significant differences, those confidence intervals that do not contain zero.

**Table S6.** Network properties (AUC) statistics and Targeted attack study comparing both age groups using Brainnetome atlas parcellation (246 anatomical structures).

| Network Property | Group 1  (Young Age) | Group 2  (Old Age) | Confidence Interval (95%) |
| --- | --- | --- | --- |
| Clustering Index | 10.80(0.22) | 11.46(0.23) | **(-1.49,-0.28)** |
| Characteristic Path Length | 29.90(0.30) | 31.24(0.52) | **(-2.82,-0.39)** |
| Local Efficiency | 13.84 (0.09) | 14.13(0.11) | **(-0.68,-0.13)** |
| Global Efficiency | 11.02 (0.07) | 10.82 (0.09) | **(0.03,0.49)** |
| Global Connectivity | 0.42(0.038) | 0.43 (0.04) | (-0.10,0.10) |
| Targeted Attack | 15.48(0.35) | 15.63 (0.35) | (-1.21,0.72) |
| Homologous regions connectivity | 0.69 (0.02) | 0.70 (0.03) | (-0.08,0.06) |

Network properties in each group are represented by the mean and standard deviations: mean (Standard deviation).

In bold the significant differences, those confidence intervals that do not contain zero.

**Table S7.** Network properties (AUC) statistics and Targeted attack study comparing both age groups using Gordon atlas parcellation (333 anatomical structures).

| Network Property | Group 1  (Young Age) | Group 2  (Old Age) | Confidence Interval (95%) |
| --- | --- | --- | --- |
| Clustering Index | 10.74 (0.31) | 11.57 (0.28) | **(-1.94,-0.20)** |
| Characteristic Path Length | 29.58 (0.25) | 30.63 (0.55) | **(-3.27,-0.62)** |
| Local Efficiency | 13.74(0.13) | 14.13(0.14) | **(-0.83,-0.06)** |
| Global Efficiency | 11.03 (0.08) | 10.83(0.13) | **(0.01,0.62)** |
| Global Connectivity | 0.33 (0.03) | 0.38 (0.032) | (-0.136,0.05) |
| Targeted Attack | 14.77 (0.49) | 14.72(0.51) | (-1.38,1.41) |
| Homologous regions connectivity | 0.38(0.032) | 0.42 (0.032) | (-0.14,0.04) |

Network properties in each group are represented by the mean and standard deviations: mean (Standard deviation).

In bold the significant differences, those confidence intervals that do not contain zero.

**Supplementary Study 1:** *Evaluation of global myelin effect differences between groups and sliding windows.*

In order to study whether exist different influences of the global myelin density (as global effect) between groups over local myelin density we performed several steps in our analysis:

1. Regressing out the Age, Gender, Age^2^, Age*Gender for all structures and global mean MT.
2. Computation of the Pearson correlation between corrected structures MT and corrected Global MT (global-local MT correlations).
3. The global-local MT correlation for each structure was statistical compared between groups; multiple comparison correction is applied. In order to compare correlation coefficients ($r_{1}$and$r_{2}$) we followed the standard procedure of converting the correlation to ‘z’ values using Fisher’s r-to-z transformation. We used the ‘Z’ statistic to compare the transformed z values in order to define the significance of the group differences correlations. The Z statistic is calculated by:$Z=\left(z_{1}-z_{2}\right)/\sqrt{1/\left(N_{1}-3\right)+1/\left(N_{2}-3\right)}$, where $N_{1}$ and $N_{2}$ are the number of data used to calculate the correlation coefficients $r_{1}$and $r_{2}$ transformed to $z_{1}$ and $z_{2}$ using the well-known ‘r’ to ‘z’ Fisher transformation $z_{i}=0.5 \ln\left(1+r_{i}\right)/\left(1-r_{i}\right)$. To adjust for multiple comparisons, a false discovery rate (FDR) procedure was performed at q value of 0.05.
4. Computation of the overall global-local MT correlation r_mean_ for each group, taking the mean over all z_i_ to calculate z_mean_ and transforming back to correlation using the inverse Fisher transformation. Statistical comparison between groups of the overall global-local MT correlation using Z statistic defined in step 3.
5. For the sliding window study a pairwise statistical comparison of the overall global-local MT correlations across all pairs of windows is performed.

Results:

As it is observed in Figure S5 panel A) only three structures showed differences in Global-Local MT correlation between groups. The overall differences was not significant p=0.82.


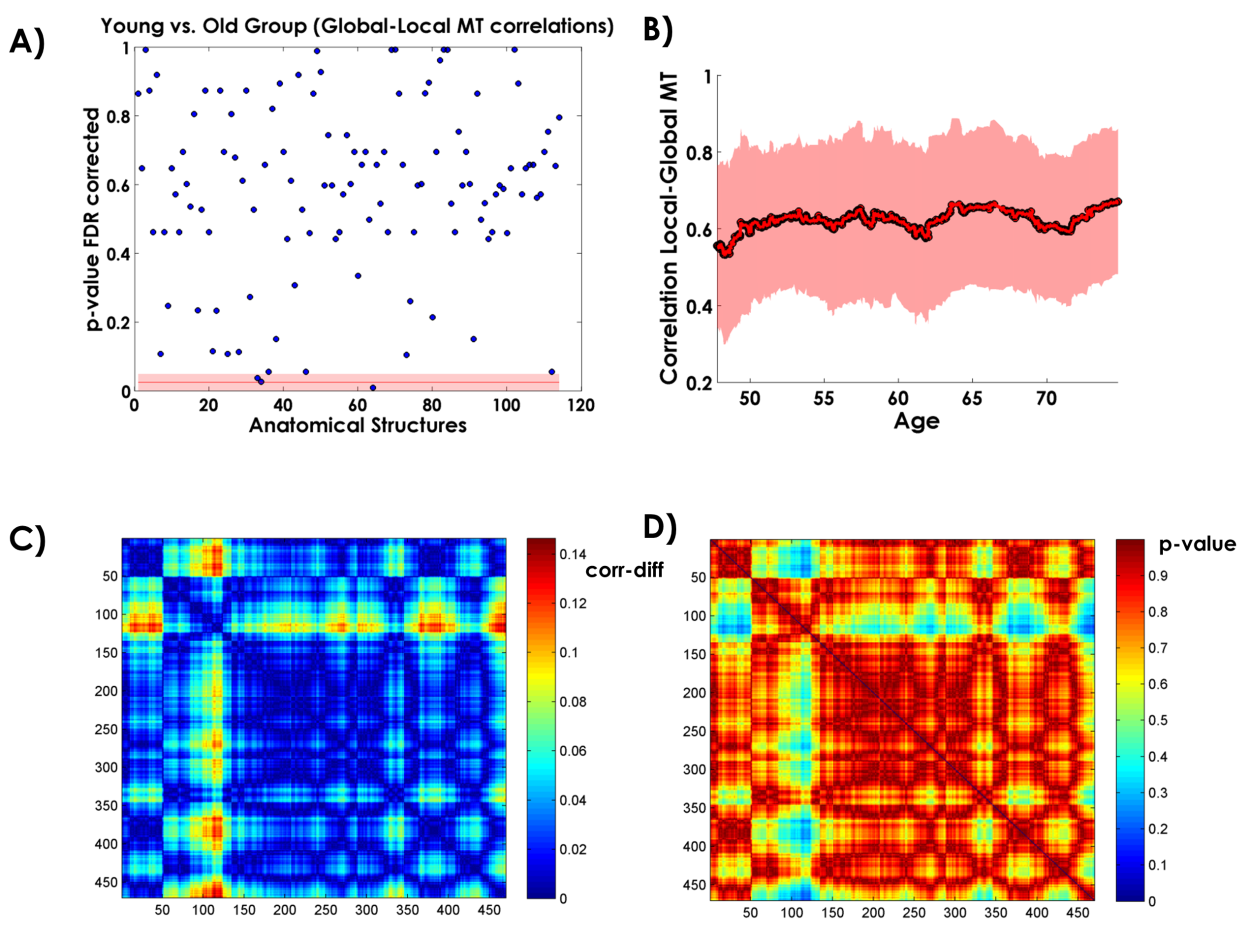


**Figure S5 A)** Differences in the Global MT effects over regional MT measured through differences in the correlation coefficient between Global MT - Local MT. Red shaded area shows the p-value<=0.05, line in red indicates p-value=0.025. **B)** Mean Global-Local MT correlation for different time windows. The red shaded area represents the standard deviation; the line in red indicates the mean across all structures. **C)** Global-Local MT correlations differences for all pairs of sliding windows **D)** p-values of the differences in Global MT -Local MT correlations for all pairs of sliding windows.

For the case of the sliding window approach no differences were found in the overall Global-Local MT correlation between any pair of windows. Figure 4.1 panel B) shows the mean correlation coefficient across for all time windows. To assess the mean correlation r_mean_ first the correlation coefficients were transformed to z, using Fisher transformation, the z values averaged in z_mean_ and finally transformed back using inverse Fisher transform to obtain r_mean_. The mean Global-Local MT correlation is around 0.6 for all sliding windows (Panel B). Panel C) shows the differences in the Global-Local MT correlation for all pairs of sliding windows. Finally panel D) depicts the p-values of the differences. These results show that there were no differences in Global-Local MT correlations. Therefore considering the ‘real physiological’ global MT as an additive effect, the corresponding aging trajectories of the global connectivity and correlation strength between homologous regions will be biased in a constant. It is expected that the rest of network attributes are less affected, since a constant added in the myelin correlation matrices wont affected the binarized graphs.
